# GC-MS based bioactive profiling of *Phyllanthus niruri* and its antibacterial potential through experimental and computational studies

**DOI:** 10.1371/journal.pone.0340866

**Published:** 2026-01-23

**Authors:** Zubair Khalid Labu, Samira Karim, Maria Afroz, Umme Kulsum Batul, Lina Akther, Md. Tarekur Rahman, Sarder Arifuzzaman

**Affiliations:** Department of Pharmacy, World University of Bangladesh (WUB), Uttara, Dhaka, Bangladesh; Jadavpur University, INDIA

## Abstract

**Objective:**

The study aimed to evaluate the antibacterial potential of *Phyllanthus niruri* by identifying its bioactive compounds through GC–MS, assessing their in vitro antibacterial efficacy, and validating their interactions with bacterial target proteins through molecular docking and pharmacokinetic analyses.

**Methods:**

Methanolic extracts were prepared and fractionated into petroleum ether (PSF), chloroform (CSF), carbon tetrachloride (CTF), ethyl acetate (ESF), methanol (MSF), and aqueous (AQF) fractions using the Kupchan method. Phytochemical screening, total phenolic content (TPC), and total flavonoid content (TFC) were determined spectrophotometrically. GC–MS analysis identified volatile constituents in the methanol extract. Antibacterial activity was evaluated against nine bacterial strains using the disc diffusion assay, Minimum Inhibitory Concentration (MIC), and Minimum Bactericidal Concentration (MBC) tests. Molecular docking (PyRx), ADMET (pkCSM), and drug-likeness (SwissADME) analyses were performed to assess pharmacological suitability.

**Results:**

Phytochemical screening confirmed the presence of major secondary metabolites such as flavonoids, tannins, and phenolics. The methanol fraction (MSF) exhibited the highest TPC (119.10 ± 0.11 µg GAE/g) and TFC (128.01 ± 0.11 µg QE/g), followed by the ethyl acetate fraction (TPC = 102.06 ± 0.11 µg GAE/g; TFC = 109.09 ± 0.21 µg QE/g). GC–MS profiling revealed 75 compounds, including 3,4-dimethoxy-dl-phenylalanine (13.24 µg/mL), benzeneacetamide (3,4-dimethoxy-, 13.24 µg/mL), and 3-(3,4-dimethoxyphenyl)-propionic acid (13.24 µg/mL).

*In vitro* assays demonstrated that the methanolic and ethyl acetate fractions exhibited the strongest antibacterial activity, with inhibition zones of 33.2 ± 0.96 mm and 15.1 ± 0.52 mm against *Escherichia coli* and *Staphylococcus aureus*, respectively. MIC values ranged from 62.5 µg/mL to 250 µg/mL, and MBC/MIC ratios ≤ 4 confirmed potent bactericidal activity. Molecular docking revealed strong ligand protein affinities, with benzeneacetamide (–9.4 kcal/mol) and 3-(3,4-dimethoxyphenyl)-propionic acid (–8.7 kcal/mol) showing the highest binding energies toward DNA gyrase and penicillin-binding protein 1B (PBP1B). ADMET and SwissADME analyses indicated favorable gastrointestinal absorption, no hepatotoxicity, and compliance with Lipinski’s rule of five.

**Conclusion:**

*Phyllanthus niruri*, particularly its polar fractions, possesses potent antibacterial phytochemicals validated through GC–MS, *in vitro*, and *in silico* studies. These findings establish its potential as a promising natural source for the development of novel antimicrobial drugs.

## 1. Introduction

**Fig 1:**
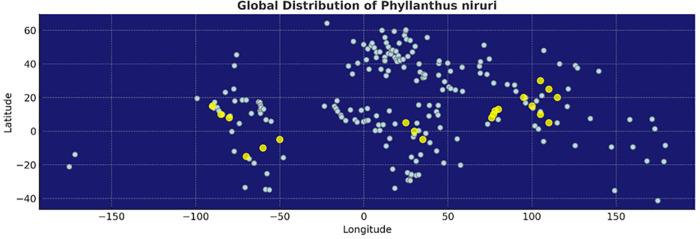
Global distribution of *Phyllanthus niruri.* Yellow markers indicate reported occurrences across Southeast Asia, Southern India, China, Africa, and the Americas. The map was created using Natural Earth public domain data (https://www.naturalearthdata.com/), which is freely available under the CC0 license.

Plant-based natural compounds have been widely used around the world as complementary and alternative therapies, playing a pivotal role in maintaining and improving human health and condition. Historically, many pharmaceutical drugs like aspirin, digoxin, morphine, ephedrine, and quinine have originated from medicinal plants, which reinforces the therapeutic potential of botanical sources. Among such plants*, Phyllanthus niruri* L., belonging to the genus Phyllanthus and the family Euphorbiaceae, has gained attention for its rich ethnomedicinal history and diverse pharmacological effects [1]. The genus Phyllanthus comprises over 600 species of shrubs, trees, and annual or biennial herbs widely distributed across tropical and subtropical regions (Fig 1). Among them, *Phyllanthus niruri* is a small, erect annual herb growing up to 30–40 cm in height, indigenous to the Amazon rainforest as well as tropical areas such as Southeast Asia, southern India, and China [2,3]. The leaf of this plant are alternate, sessile, oblong, and elliptic, measuring 7–12 cm in length, and are arranged distichously, often imbricating. It bears small, off-white to greenish flowers that are solitary, axillary, pedicellate, apetalous, and monoecious [4], with male flowers occurring in groups of one to three and female flowers occurring singly. Male flowers are yellow-white with a reddish tinge at the base and typically possess six sepals or petals [5]. The anthers are yellow, measuring 0.25–0.4 mm in length and 0.3–0.45 mm in width. The capsules are oblate, stramineous, and approximately 3 mm in diameter, with clearly visible venation and very acute stipules [6,7]. It also has a long-standing role in traditional herbal medicine systems such as Ayurveda, Traditional Chinese Medicine, and Indonesian Jamu [4]. In India, where it is referred to as Pitirishi or Budhatri, it is a common household remedy for conditions such as asthma, bronchitis, anaemia, tuberculosis, coughs, jaundice, and extreme thirst [8]. The whole plant is used to treat various ailments including dysentery, vaginitis, influenza, tumours, diabetes, dyspepsia, kidney stones, hepatitis B, and hyperglycaemia, and is recognized for its diuretic, hepatoprotective, and antimicrobial properties [9]. In Ayurvedic medicine, Phyllanthus niruri has also been used for over 2000 years for conditions such as jaundice, gonorrhoea, frequent menstruation, and diabetes [4].

The therapeutic efficacy of this plant is attributed to its rich phytochemical profile, including lignans, tannins, coumarins, terpenes, flavonoids, alkaloids, saponins, and phenylpropanoids, which are distributed throughout the plant’s leaf, stems, and roots. It also contains common lipids, sterols, and flavonols [8]. To gain insight into these bioactive phytoconstituents, modern analytical techniques such as Gas Chromatography–Mass Spectrometry (GC–MS) have become essential tools. GC–MS enables the rapid identification and characterization of volatile and semi-volatile compounds in complex plant extracts with high precision and sensitivity [9]. In this study, GC–MS was employed to identify key bioactive compounds in the methanolic extract of leaf, providing a chemical fingerprint that supports further pharmacological analysis and strengthens the foundation for therapeutic validation. However, the pharmacological potential of plant leaf has been confirmed through various clinical and preclinical studies. Pharmacological studies have documented the diuretic, hypotensive, and hypoglycaemic activities of this plant [10]. Clinical evaluations have demonstrated that *Phyllanthus niruri* significantly increases diuresis, reduces systolic blood pressure in hypertensive individuals, and lowers blood glucose levels in diabetic patients [11,12]. Despite its wide usage, scientific evaluation of these leaf is still fragmented, with a lack of consolidated evidence from both clinical and mechanistic studies. The variability of primary research and limited high-throughput screening data have slowed down its progress as a validated therapeutic agent. The alarming rise of multidrug-resistant bacteria has intensified the search for alternative antimicrobial agents with novel mechanisms of action. Medicinal plants, including *Phyllanthus niruri*, represent promising sources of bioactive compounds with therapeutic potential but remain underexplored for antibacterial activity. Integrating *in vitro* and *in silico* approaches can accelerate the identification of effective natural compounds for future drug discovery.

Nonetheless, its potential remains promising, especially in the era of rising antimicrobialresistance, which has rendered many conventional antibiotics less effective and led to an urgent global demand for novel antimicrobial agents. In this context, natural sources such as *Phyllanthus niruri* offer a less toxic and economically viable alternative to synthetic drugs.

However, despite its rich phytochemical profile and traditional medicinal use, there is still limited understanding of the specific bioactive compounds responsible for its antimicrobial effects and their molecular targets. Given the emerging value of computational tools in drug discovery, our study aimed to investigate both the *in vitro* and *in silico* antimicrobial potential of leaf extracts. Through this dual approach, we not only assessed its biological efficacy in laboratory conditions but also explored molecular-level interactions with target proteins relevant to microbial infections. While previous studies have reported the antibacterial activity of *Phyllanthus niruri,* most have focused either on crude extract testing or general GC–MS characterization without correlating specific identified compounds to bacterial target proteins. In contrast, our study establishes this link by selecting GC–MS–identified molecules with literature-based antimicrobial relevance, performing molecular docking against validated bacterial enzymes and integrating pharmacokinetic predictions to assess their translational drug potential.

## 2. Materials and methods

### 2.1. Sampling and proper documentation

Fresh leaf of *Phyllanthus niruri* were collected from Dhaka, in the month of February,2024. The plant specimen was taxonomically authenticated by an expert taxonomist at the Bangladesh National Herbarium, Mirpur, Dhaka, where it was assigned the accession number [2347RT23]. Prior to collection, necessary permissions and ethical approvals were obtained to ensure compliance with institutional and national regulatory standards. Collected specimens were properly documented, labelled, and preserved for subsequent analyses conducted in this study.

### 2.2. Preparation of *Phyllanthus niruri* plant extract

After collection, the plant leaf were thoroughly washed with distilled water to remove dirt, dust, and other contaminants. The cleaned leaf were initially air-dried under shade for 7 days, followed by further drying in a mechanical hot air oven (Memmert UN55, Germany) at 35–40 °C to ensure complete removal of moisture. The completely dried leaf were ground into a coarse powder using a mechanical grinder (Philips HL7756/00 Mixer Grinder, India). The powdered plant material was stored in an airtight amber glass container at room temperature (25 ± 2 °C) in a cool, dry, and dark place to prevent degradation of bioactive constituents prior to extraction and analysis.

For extraction, 400 g of the powdered plant material was transferred into a clean, flat-bottomed amber glass container contained in 2000 mL (1:5 ratio) of methanol at 25°C. The extraction process was allowed to stand and macerate for 7 days, with occasional stirring, until the siphoning solvent colourless, indicating thorough extraction. The resulting methanolic extract was filtered first using cotton and then through Whatman No. 1 filter paper. The filtered extract was concentrated using a rotary evaporator with a water bath maintained at 40°C under reduced pressure to 100mbar. The rotary evaporator was operated at a rotation speed of 120 rpm, and the condenser temperature was maintained between –5°C to 5°C to ensure efficient condensation and recovery of methanol vapours. A total of 10 g of the crude extract was subjected to successive solvent-solvent partitioning, yielding various fractions based on their increasing polarity, following the standard Kupchan partitioning method [13,14]. The process resulted in the separation of the ethyl acetate-soluble fraction (ESF, 1.9 g), petroleum ether-soluble fraction (PSF, 0.7 g), chloroform-soluble fraction (CSF, 0.6 g), carbon tetrachloride-soluble fraction (CTF, 0.8 g), methanol-soluble fraction (MSF, 3.5 g), and aqueous fraction (AQF, 2.5 g). These fractions were then concentrated and stored for further phytochemical and pharmacological evaluations The Kupchan partitioning method is a strategic approach to simplify crude extracts, enhance bioactivity screening, and facilitate compound isolation. By fractionating plant extracts into polarity-based groups, this method significantly contributes to natural product research, drug discovery, and pharmacological studies [15].

### 2.3. Chemicals and reagents

Analytical-grade chemicals were used throughout the study to ensure precision and consistency in all experimental procedures. The reagents employed included methanol (liquid chromatography grade, ≥ 99.8%), ethyl acetate (≥99.9% GC), petroleum ether (≥80%), chloroform (≥99% ACS reagent grade), carbon tetrachloride (≥99.9%), gallic acid (98%), catechin (≥99.8%), Folin-Ciocalteu reagent (standard reagent grade), and aluminium chloride (anhydrous, sublimed, ≥ 99.8%). All chemicals were sourced from Science Park Chemicals Ltd., Bangladesh, and handled according to standard laboratory safety protocols.

### 2.4. Phytochemical screening of plant extract

#### 2.4.1. Phytochemical screening of crude extracts.

A qualitative phytochemical screening was performed to identify the presence of different bioactive constituents in all crude solvent extracts of *Phyllanthus niruri*. Standard phytochemical tests were carried out following slightly modified protocols [16]. These tests included Molisch’s test, Fehling’s solution test, Lead acetate test, Ferric chloride test, Mayer’s and Wagner’s tests, Shinoda test, Frothing test, and Libermann-Burchard’s test. The results confirmed the presence of major phytochemical groups such as carbohydrates, reducing sugars, tannins, alkaloids, flavonoids, saponins, steroids, phenols, and glycosides across various solvent fractions. All observations were carefully recorded and presented in Table 1.

**Table 1:** Preliminary phytochemical screening of crude methanolic extract of *Phyllanthus niruri* leaf extract.

Phytochemicals	Name of the test	Remarks
Carbohydrates	Molisch’s Test	+
Reducing sugars	Fehling solution test	+
Tannins	Lead acetate test	+++
Alkaloids	Mayer’ s test	++
Flavonoids	Shinoda test	+++
Saponins	Frothing test	+++
Steroids	Libermnn-Burchards test	+
Phenol	Ferric chloride test	+++

Amounts shown as + = Mildly present, ++ = Moderately present, +++ = Largely present, depending on the depth of colour.

#### 2.4.2. Estimation of total phenolic content (TPC).

The total phenolic content was assessed following the procedure outlined by Velioglu *et* al. [17]. In this method, 1 mL of the crude methanolic extract and its respective fractions (at a concentration of 1000 μg/mL) each were combined with 1 mL of Folin-Ciocalteu reagent in separate test tubes. After allowing the reaction to proceed for 5 minutes, 10 mL of a 7% sodium carbonate (Na₂CO₃) solution was added, followed by 13 mL of deionized distilled water to bring the total volume to the desired level. The mixtures were thoroughly mixed and incubated in a dark environment at 23°C for 90 minutes. For standardization, gallic acid solutions with concentrations of 62.5, 125, 250, 500, and 1000 μg/mL were prepared using the same protocol. The absorbance of each sample was then recorded at 750 nm using a spectrophotometer, with methanol serving as the blank control. The TPC was expressed in terms of gallic acid equivalents (GAE) and calculated using the following equation:


C = (c × V) / m 

where C is the total phenolic content (mg GAE/g of extract), c represents the gallic acid concentration determined from the standard curve (mg/mL), V is the volume of the extract (mL), and m denotes the dry weight of the extract (g).

#### 2.4.3. Estimation of total flavonoid content (TFC).

The total flavonoid content was evaluated using the method developed by Nicolescu *et* al. [18]. In this assay, 0.3 mL of the crude methanolic extract and its respective fractions from *Phyllanthus niruri* were mixed with 3.4 mL of 30% methanol, 0.15 mL of 0.3 M aluminum chloride hexahydrate (AlCl₃·6H₂O), and 0.15 mL of 0.5 M sodium nitrite (NaNO₂) in separate test tubes. After an incubation period of 5 minutes, 1 mL of 1 M sodium hydroxide (NaOH) was added to each tube. The mixture was then thoroughly shaken to ensure complete reaction. The absorbance of the resulting solution was recorded at 506 nm using a UV-visible spectrophotometer, with reagent blanks serving as the control. For calibration, catechin solutions were prepared at concentrations of 62.5, 125, 250, 500, and 1000 μg/mL, following the same procedure as used for the sample extracts. The total flavonoid content was expressed in catechin equivalents (CE) and calculated using the equation:


 C = (c × V) / m

where C represents the flavonoid content (mg CE/g of extract), c is the catechin concentration obtained from the standard curve (mg/mL), V is the volume of the extract (mL), and m is the dry weight of the extract (g).

#### 2.4.4. GC-MS Sample Preparation and Analysis.

To prepare the sample for GC-MS analysis, 10 mg of the dried methanolic extract of leaf was dissolved in 1 mL of GC-MS-grade methanol. The solution was thoroughly vortexed and centrifuged to remove any insoluble particles. The clear supernatant obtained was carefully collected and filtered using a 0.22 µm syringe filter to ensure sample clarity. Subsequently, 1 µL of the filtered solution was injected into the GC-MS instrument for analysis.

GC–MS analysis was carried out using an Agilent 7890A capillary gas chromatograph (Agilent Technologies), coupled with a 5975C inert XL EI/CI triple-axis mass detector. Separation was performed on an HP-5MSI fused silica capillary column (5% phenyl, 95% dimethylpolysiloxane; 0.25 μm film thickness, 90 m length, 0.25 mm internal diameter).

The chromatographic conditions were as follows: the oven temperature was initially set at 90°C (held for 0 min), ramped to 200°C at 3°C/min and held for 2 min, then increased to 280°C at 15°C/min and held for 2 min. The total run time was 50 minutes. Helium was used as the carrier gas at a flow rate of 1.1 mL/min. The inlet temperature was maintained at 250°C, and the auxiliary temperature was set at 280°C.

Mass spectrometric conditions included a quadrupole temperature of 150°C, ion source temperature of 230°C, and mass scan range of 50–550 m/z in scan mode. Identification of compounds was achieved by comparing the mass spectra with entries in the NIST-MS Library. The relative percentage of each identified compound was determined based on the peak areas in the total ion chromatogram (TIC).

To ensure analytical reliability, compound identification was validated by comparing mass spectra with multiple entries in the NIST 2020 and Wiley libraries. Only compounds with a match quality ≥90% were considered. Quantitative confirmation was performed using peak area normalization. The novelty of major compounds was evaluated through PubChem and existing phytochemical databases to determine whether they had been previously reported in *Phyllanthus niruri.*

### 2.5. *In-vitro* study design for antimicrobial evaluation

#### 2.5.1. Test organisms for antimicrobial evaluation.

The bacterial strains used in this study were collected from the Department of Microbiology, University of Dhaka. The gram-positive bacterial strains included *Bacillus subtilis*, *Bacillus cereus*, *Staphylococcus aureus*, and *Sarcina lutea*, while the gram-negative strains consisted of *Escherichia coli*, *Salmonella typhi*, *Vibrio mimicus*, *Vibrio parahaemolyticus*, and *Bacillus parahaemolyticus*. All bacterial cultures were maintained at a constant temperature of 37°C prior to use in antimicrobial testing.

#### 2.5.2. Preparation of inoculum.

To prepare the bacterial inoculum, 5 mL of nutrient broth was dispensed into sterilized test tubes, sealed with cotton plugs, and autoclaved for complete sterilization. Once cooled, the test tubes were labelled accordingly and arranged on a slide rack. Each tube was then inoculated with two loopfuls of individual bacterial strains comprising a total of nine organisms, including five gram-negative and four gram-positive bacteria. The contents were thoroughly mixed and incubated at 37°C for 18–24 hours. Following incubation, visible microbial growth confirmed successful inoculation.

#### 2.5.3. Antibacterial activity.

The antibacterial potential of leaf extracts was evaluated using the disc diffusion method. Various solvent fractions (CTF, PSF, ESF, MSF, and CSF) were tested at 1000 µg/disc against Gram-positive strains (*Bacillus subtilis* ATCC 6633, *Bacillus cereus* ATCC 14579, *Staphylococcus aureus* ATCC 25923, *Sarcina lutea* ATCC 9341) and Gram-negative strains (*Escherichia coli* ATCC 25922, *Salmonella typhi* ATCC 19430, *Vibrio mimicus* ATCC 33653, *Vibrio parahaemolyticus* ATCC 17802, *Bacillus parahaemolyticus* ATCC 33844). Sterile filter paper discs were impregnated with the extracts and placed on nutrient agar plates pre-seeded with the test bacteria. Plates were first kept at 4°C for 24 hours to allow diffusion, then incubated at 37°C for another 24 hours. Ciprofloxacin (5 µg/disc; Che-5) and methanol (40 µL/disc) served as positive and negative controls, respectively. All tests were performed in triplicate, and inhibition zones were measured (mm) and expressed as mean ± SD to determine antibacterial activity [19,20]. The selected bacterial strains represent clinically relevant pathogens responsible for various infectious diseases.

#### 2.5.4. Minimum inhibitory concentration (MIC).

The MIC of the leaf extracts were determined using the broth microdilution method as described by Wiegand *et* al. and Balouiri *et* al. [21,22], with minor modifications. Serial two-fold dilutions of each extract and its solvent fractions (CTF, PSF, ESF, MSF, and CSF) were prepared in Mueller-Hinton broth (MHB) to obtain concentrations ranging from 1000 µg/mL to 7.8 µg/mL. Each tube received 1 mL of standardized bacterial suspension (approximately 1 × 10⁶ CFU/mL) prepared from 24-hour-old cultures adjusted to 0.5 McFarland standard.

The antibacterial activity was tested against both Gram-positive and Gram-negative bacteria, including *Bacillus subtilis*, *Bacillus cereus*, *Staphylococcus aureus*, *Sarcina lutea* (Gram-positive), and *Escherichia coli*, *Salmonella typhi*, *Vibrio mimicus*, *Vibrio parahaemolyticus*, and *Bacillus parahaemolyticus* (Gram-negative). Ciprofloxacin (5 µg/disc; Che-5) was used as a positive control, and methanol (40 µL/disc) served as the negative control. The tubes were incubated at 37°C for 18–24 hours under aerobic conditions. After incubation, the tubes were visually examined for turbidity or pellet formation indicating bacterial growth. The lowest concentration showing no visible growth was recorded as the MIC. To verify results, optical density readings at 600 nm were measured using a UV visible spectrophotometer (Shimadzu UV-1800, Japan).

#### 2.5.5. Minimum bactericidal concentration (MBC).

The Minimum Bactericidal Concentration (MBC) test was performed to determine the lowest concentration *of* leaf extract and its solvent fractions (CTF, PSF, ESF, MSF, and CSF) that could kill the bacterial cells, rather than merely inhibiting their growth. The MBC assay was conducted following the procedures outlined by Wiegand *et* al. and Balouiri et al. [21–23], with necessary modifications to fit plant extract analysis.

After determination of the MIC, all tubes or wells that showed no visible growth or turbidity were selected for MBC testing. From each of these tubes, 0.1 mL aliquots were aseptically withdrawn using sterile micropipette tips and spread-plated onto freshly prepared nutrient agar plates that contained no antimicrobial agents. These plates were then incubated at 37°C for 24 hours under appropriate conditions to allow any surviving bacterial cells to form visible colonies. After incubation, plates were examined for bacterial growth.

The MBC was defined as the lowest concentration of extract that produced no visible bacterial colonies on the agar surface, indicating complete elimination of viable bacterial cells. This endpoint confirms the bactericidal activity of the test substance, distinguishing it from merely bacteriostatic effects observed in the MIC assay.

To assess the bactericidal strength of each extract, the MBC/MIC ratio was calculated. If the ratio was ≤ 4, the extract was considered to exhibit bactericidal activity, while a ratio > 4 indicated bacteriostatic activity. Each experiment was performed in triplicate to ensure precision and reproducibility of the data.

The MBC test was conducted against the same Gram-positive and Gram-negative bacterial strains used in the MIC assay, including *Bacillus subtilis, Bacillus cereus, Staphylococcus aureus, Sarcina lutea, Escherichia coli, Salmonella typhi, Vibrio mimicus, Vibrio parahaemolyticus,* and *Bacillus parahaemolyticus*. Ciprofloxacin (5 µg/disc; Che-5) served as the positive control due to its well-established bactericidal activity, while methanol (40 µL/disc) was included as a negative control to rule out solvent effects.

The MBC determination provided a reliable estimation of the killing potential of *Phyllanthus niruri* extracts, offering insight into their potential as natural antibacterial agents. The inclusion of both Gram-positive and Gram-negative organisms further enabled evaluation of the broad-spectrum efficacy of the extracts.

#### 2.5.6. Statistical evaluation.

The experimental results were processed using SPSS software version 22.0 (SPSS Inc., Chicago, IL, USA). Data represent the mean ± standard deviation (SD) calculated from three independent replicates. To assess significant differences between group means, one-way ANOVA was performed followed by Duncan’s Multiple Range Test (DMRT) as a post-hoc analysis, considering p-values less than 0.05 as statistically significant.

### 2.6. *In-silico* study analysis

#### 2.6.1. Selection and preparation of ligands and standard drug.

To support and extend the *in vitro* findings, 10 major compounds selected based on their high concentration per volume of extract and known antimicrobial profiles were subjected to *in silico* analysis to identify potential bioactive constituents responsible for the experimental outcomes, as presented in Table 8. Ciprofloxacin, a fluoroquinolone antibiotic known for its broad-spectrum efficacy against both Gram-positive and Gram-negative bacteria, was employed as the standard reference drug [24,25]. Its antimicrobial action works by targeting and inhibiting bacterial enzymes DNA gyrase and topoisomerase IV, which are critical for processes such as DNA replication, transcription, repair, and recombination. Inhibiting these enzymes interferes with bacterial DNA functions, ultimately causing cell death and effective microbial clearance [26]. The three-dimensional (3D) structures of all selected ligands, along with ciprofloxacin, were downloaded in Structure Data File (SDF) format from the PubChem database (https://pubchem.ncbi.nlm.nih.gov/). For proper docking analysis, the molecular structures were prepared and geometrically optimized. This involved ligand preparation using Gaussian and GausView software, followed by energy minimization using Gabedit to ensure the ligands were in their most stable conformations for computational modelling.

#### 2.6.2. Selection and preparation of target proteins.

Based on the *in vitro* findings, where four Gram-positive and five Gram-negative bacterial strains were tested against five different fraction of plant extracts, *Staphylococcus aureus* (Gram-positive) and *Escherichia coli* (Gram-negative) were selected for further *in silico* studies due to their consistently strong susceptibility across all extract types. To investigate the molecular interactions responsible for this antimicrobial activity, six target proteins were selected through literature review. For *Staphylococcus aureus*, the chosen targets included Penicillin-Binding Protein 1B (PBP1B) [PDB ID: 2Y2I], Isoleucyl-tRNA synthetase (IleRS) [PDB ID: 1FFY], DNA Gyrase Subunit B (GyrB) [PDB ID: 3G75], and Penicillin-Binding Protein 3 (PBP3) [PDB ID: 3VSL] [27,28]. For *Escherichia coli*, DNA Gyrase Subunit B (GyrB) [PDB ID: 4PRX] and the Catalytic α-subunit of DNA Polymerase III (Pol III α-subunit) [PDB ID: 2HNH] were selected [29,30]. The 3D structures of these proteins were retrieved from the RCSB Protein Data Bank (https://www.rcsb.org/) and visualized using Discovery Studio software. During preparation, all non-essential molecules were removed, and the refined protein structures were saved in PDB format (Fig 5). To ensure structural stability and accuracy, each protein was then subjected to energy minimization using Swiss-PDB Viewer (version 4.1.0).

Docking reliability was strengthened by revalidating each target protein using redocking of the co-crystallized ligand, ensuring RMSD ≤ 2.0 Å. Grid box parameters were optimized around active sites identified from crystallographic data. Binding energies were compared to ciprofloxacin as a positive control to assess relative binding strengths.

#### 2.6.3. Molecular docking and non-bonding interaction analysis.

To elucidate the molecular basis of the observed antimicrobial activity, molecular docking simulations were systematically carried out to quantify the binding affinities between selected phytochemicals and bacterial protein targets. Using PyRx (version 0.8), a total of 10 plant-derived compounds, alongside the standard antibiotic ciprofloxacin, were docked against structurally characterized targets from both Gram-positive and Gram-negative bacterial strains. The docking scores, presented in Table 9, provided a comparative framework to evaluate ligand protein interactions. Among these, the ligands exhibiting the lowest binding energy (i.e., strongest affinity) across multiple targets were prioritized for in-depth interaction profiling, as listed in Table 10.

Subsequent structural visualization was conducted using PyMOL, which facilitated the identification and extraction of binding poses in PDB format. These docked complexes were further analyzed using Discovery Studio to generate both 2D and 3D interaction maps. Figs 7–18 illustrate the spatial orientation and types of non-covalent interactions (e.g., hydrophobic, van der Waals, and electrostatic), while Figs 19–24 specifically highlights the hydrogen bonding patterns between ligands and amino acid residues within the active sites.

The interaction profiles were critically examined to identify key residues involved in stabilizing the ligand–receptor complexes, revealing consistent binding motifs that may underlie the antimicrobial efficacy. These findings, summarized in Table 11, provide molecular-level insight into the binding preferences of each compound and offer a rationale for their biological activity, thereby guiding future optimization of lead candidates.

#### 2.6.4. ADMET study analysis.

The compound identified through molecular docking was further evaluated for its pharmacokinetic properties, specifically focusing on absorption, distribution, metabolism, excretion, and toxicity (ADMET). For this purpose, the molecular structures were converted into SMILES format and analysed using the pkCSM web server (http://biosig.unimelb.edu.au/pkcsm/prediction). This tool provided comprehensive predictions for key ADMET parameters, offering valuable insights into the compound’s potential behaviour within biological systems [31]. Compounds demonstrating favourable pharmacokinetic profiles were considered promising candidates and prioritized for subsequent analysis.

#### 2.6.5. Drug likeness study analysis.

To assess its drug-likeness profile, the selected compound was further analysed using the Swiss ADME tool (http://www.swissadme.ch/). The structure was first converted into canonical SMILES format and then evaluated for various *in silico* pharmacokinetic properties, including physicochemical characteristics, hydrophilicity, lipophilicity, and bioavailability. The analysis was guided by Lipinski’s Rule of Five, a standard criterion for predicting the oral bioavailability of new molecular entities (NMEs). According to this rule, a compound is less likely to have good absorption or permeability if it has more than five hydrogen bond donors, more than ten hydrogen bond acceptors, a molecular weight over 500 Daltons, or a Log P value greater than 5. Compounds violating more than two criteria are generally considered less favourable for oral administration [32–34].

## 3. Result and discussion

### 3.1. Analysis of phytoconstituents of crude methanol extract

Preliminary phytochemical screening of the crude methanolic extract of leaf revealed the presence of several bioactive constituents, including steroids, tannins, saponins, phenols, and flavonoids. Among these, phenols, saponins, and flavonoids showed particularly strong results in the qualitative analysis (Table 1). This was further supported by the quantitative analysis, which confirmed a substantial presence of these compounds (Table 2). These phytochemicals are well-documented for their broad therapeutic potential, including applications in the treatment of diabetes, obesity, cancer, and cardiovascular diseases [35]. In particular, flavonoids and phenolic compounds are recognized for their potent antimicrobial activity, which likely contributes to the biological effects observed in this study [36].

**Table 2:** Total phenolic and total flavonoid contents of various extractives leave of *Phyllanthus niruri.*

Sample	Polarity	TPC (µg of GAE/g of extract)	TFC (µg of CE/g of extract)
CTF	Non-polar	60.11 ± 0.2	100.31 ± 0.23
PSF	Non-polar	51.21 ± 0.55	101.01 ± 0.14
CSF	Non-polar	70.12 ± 0.15	89.20 ± 0.14
ESF	Moderately polar	102.06 ± 0.11	109.09 ± 0.21
MSF	Polar protic	119.10 ± 0.11	128.01 ± 0.11
AQSF	Highly polar	111.03 ± .01	123.17 ± .69

CTF = Chloroform Fraction; PSF = Petroleum Ether Fraction; CSF = Carbon Tetrachloride Fraction; ESF = Ethyl Acetate Fraction; MSF = Methanol Fraction; AQSF = Aqueous Fraction.

TPC expressed as micrograms of gallic acid equivalents (GAE) per gram of extract, TFC expressed as micrograms of quercetin equivalents (QE) per gram of extract. Values are presented as mean ± standard deviation (n = 3).

### 3.2. Estimation of total phenolic contents (TPC) and total flavonoid content (TFC)

TPC and TFC of *Phyllanthus niruri* extracts were quantified in various solvents with differing polarities (Table 2). The solvents are arranged in order of increasing polarity, ranging from non-polar to highly polar (distilled water). Our results indicate a clear trend in both TPC and TFC values with respect to solvent polarity. Non-polar solvents such as PSF, CSF and CTF yielded the lowest TPC (51.21 ± 0.55, 60.11. ± 0.27 and 70.12 ± 0.15 µg GAE/g extract, respectively) and TFC (101.01 ± 0.14, 100.31 ± 0.14and 89.20 ± 0.14 µg QE/g extract, respectively). As solvent polarity increased, both phenolic and flavonoid extraction efficiencies improved markedly. ESF, a moderately polar solvent, extracted 102.06 ± 0.11 mg GAE/g TPC and 109.09 ± 0.21 µg QE/g TFC. Among all the solvents tested, methanol a polar protic solvent demonstrated the highest extraction efficiency, yielding TPC and TFC values of 119.10 ± 0.11 µg GAE/g and 128.01 ± 0.11 µg QE/g, respectively. This indicates that methanol was the most effective solvent for extracting phenolic and flavonoid compounds from *Phyllanthus niruri*. The AQSF also showed a high TFC and TPC value of 123.17 ± 0.69 and 111.03 ± .01 µg QE/g respectively, which is consistent with the findings of Edziri et al., supporting the use of polar solvents for maximizing the recovery of bioactive compounds [20]. Ethanol and distilled water, both polar protic solvents, demonstrated high extraction capabilities. These findings suggest that solvent polarity significantly influences the extraction of phenolic and flavonoid compounds from *Phyllanthus niruri*, highlighting the importance of appropriate solvent selection to maximize the recovery of bioactive constituents in phytochemical studies and underscoring their potential in managing various diseases [37]^.^ The high flavonoid content suggests a significant role in the plant’s antimicrobial activity [38], while the elevated levels of phenolic compounds may further enhance this effect [39]. These findings highlight the strong potential of *Phyllanthus niruri* as a rich source of health-promoting phytochemicals [40].

Phenolic compounds and flavonoids are two major groups of natural chemicals (secondary metabolites) found in many medicinal plants and food sources. These compounds are well known for their biological activities, especially for helping to fight infections [41]. The total phenolic content and total flavonoid content are often measured in plant extracts to estimate their medicinal potential. A higher TPC or TFC usually means the extract has more active compounds and might have stronger health benefits. These values are particularly important in developing plant-based treatments for metabolic diseases, like diabetes, and infections caused by bacteria [42,43].

Among the fractions tested, the polar protic methanolic fraction (MSF) showed the highest total phenolic content (119.10 ± 0.11 µg GAE/g) and total flavonoid content (128.01 ± 0.11 µg QE/g), indicating a higher concentration of bioactive compounds compared to other fractions. Therefore, this fraction was selected for GC–MS analysis and in silico molecular docking studies to prioritize the fraction with the greatest potential pharmacological activity. While all fractions were evaluated in in vitro assays to assess overall biological activity, focusing on MSF for chemical profiling and computational studies allowed for a more targeted investigation of the most potent bioactive constituents.

## 4. Identification and quantification of chemical compounds in methanolic extract

GC-MS analysis identified and quantified 75 compounds in the methanolic extract of *Phyllanthus niruri*. The GC-MS chromatogram illustrates the separation pattern of these compounds, while the chemical constituents along with their retention time (RT), molecular formula, molecular weight (MW), and concentration (µg/mL) in the methanol extract are detailed in Table 3. The retention times range approximately from 6.485 minutes to 32.107 minutes. This variation reflects the differences in chemical properties such as polarity, molecular size, and interactions with the stationary phase within the chromatography column. Compounds with shorter retention times (around 6–8 minutes) are generally smaller, less polar, or interact weakly with the stationary phase, allowing them to elute faster. Those with medium retention times (about 9–13 minutes) tend to have moderate polarity and size. Compounds with higher retention times (around 14–32 minutes) are usually larger or more polar, resulting in stronger retention and slower elution.

**Table 3:** List of compounds identified and semi-quantified by GC-MS analysis of the methanolic extract of *Phyllanthus niruri*. Compounds are classified based on functional group and structural features.

Sl. No.	Retention time	Conc. (ug/ml)	Compound name	IUPAC name	Molecular weight	Molecular formula	Functional group	Molecular type	Bioactivity
1.	6.485	0.343	2-Propanamine, 1-methoxy-	1-methoxypropan-2-amine	89.14 g/mol	C_4_H_11_NO	Amine group (-NH₂), Methoxy group (-OCH_3_)	Amine derivative	Inhibit papilloma virus growth, act as an agonist of toll-like receptor 4, and inhibit hepatitis C virus polymerase activity. [44]
2.	6.485	0.343	1-Propanol, 2-amino-, (. + /-.)-	2-aminopropan-1-ol	75.11 g/mol	C_3_H_9_NO	Amine group (-NH₂), Hydroxyl group (-OH)	Amino alcohol	Anti-coagulant, Antimicrobial, Antagonist of Norepinephrine [45]
3.	6.485	0.343	2-Hexanamine, 4-methyl-	4-methylhexan-2-amine	115.22 g/mol	C_7_H_17_N	Amine group (-NH₂)	Aliphatic amine	Blood Vessel Constriction, Cardiovascular Effects, Respiratory Effects [46]
4.	6.876	0.182	D-Alanine	(2R)-2-aminopropanoic acid	89.09 g/mol	C_3_H_7_NO_2_	Amine group (-NH₂), Carboxyl group (-COOH)	Amino acid	Immune Response, Gut-Microbiota-Brain Axis, Bacterial Cell Wall [47]
5.	9.167	0.396	Benzeneethanamine, N-methyl-	N-methyl-2-phenylethanamine	135.21 g/mol	C_9_H_13_N	Amine group (-NH₂)	Aromatic amine	Lipophilicity, Neurotransmitter and Receptor Interactions [46]
6.	9.167	0.396	dl-Alanine	2-aminopropanoic acid	89.09 g/mol	C_3_H_7_NO_2_	Amine group (-NH₂), Carboxyl group (-COOH)	Amino acid	Nitrogen Transport, Masking Agent, Glucose-Alanine Cycle [48]
7.	10.211	0.452	Ethanol, 2-(methylamino)-	2-(methylamino)ethanol	75.11 g/mol	C_3_H_9_NO	Amine group (-NH₂), Hydroxyl group (-OH)	Amino alcohol	Surfactant Properties, Lipid Component, CO2 Removal [49]
8.	11.118	0.461	1,2-Propanediamine	propane-1,2-diamine	74.13 g/mol	C_3_H_10_N_2_	Amine groups (-NH₂)	Diamine	Antimicrobial activity, Antioxidant activity, Potential anticancer activity [50]
9.	11.118	0.461	sec-Butylamine	butan-2-amine	73.14 g/mol	C_4_H_11_N	Amine group (-NH₂)	Aliphatic amine	Fungicidal properties, Bioaccumulation, Metabolism [51]
10.	12.045	0.381	Norephedrine, (. + /-.)-	2-amino-1-phenylpropan-1-ol	151.21 g/mol	C_9_H_13_NO	Amine group (-NH₂), Hydroxyl group (-OH)	Phenethylamine derivative	Elevates heart rate and blood pressure, releases glucose for energy, increases blood flow to muscles, and reduces blood flow to the digestive system. [52]
11.	12.889	0.303	Alanine	(2S)-2-aminopropanoic acid	89.09 g/mol	C_3_H_7_NO_2_	Amine group (-NH₂), Carboxyl group (-COOH)	Amino acid	Energy metabolism, nitrogen transport, and glucose production. [41]
12.	12.889	0.303	Meglumine	(2R,3R,4R,5S)-6-(methylamino)hexane-1,2,3,4,5-pentol	195.21 g/mol	C_7_H_17_NO_5_	Amine group (-NH₂), Hydroxyl group (-OH)	Amino sugar	Inhibition of key metabolic enzymes, induction of oxidative stress, modulation of the host immune response, and disruption of intracellular signalling in the parasites [42]
13.	13.900	0.378	Cyclobutanol	cyclobutanol	72.11 g/mol	C_4_H_8_O	Hydroxyl group (-OH)	Cycloalcohol	Antibacterial and Antimicrobial, Immunosuppressive, Antitumor [43]
14.	13.900	0.378	Tuaminoheptane	heptan-2-amine	115.22 g/mol	C_7_H_17_N	Amine group (-NH₂)	Aliphatic amine	Skin Irritation, Decongestant and Stimulant Effects [53]
15.	13.900	0.378	Decanoic acid, methyl ester	methyl decanoate	186.29 g/mol	C_11_H_22_O_2_	Ester group (-COO-)	Fatty acid ester	Antibacterial and Antifungal Activity, Anti-inflammatory Activity [54]
16.	13.900	0.378	Methyl tetradecanoate	methyl tetradecanoate	242.4 g/mol	C_15_H_30_O_2_	Ester group (-COO-)	Fatty acid ester	Flavour Enhancement and Sweetness, Metabolic Role, Nanoparticle Production [54]
17.	13.900	0.378	Hexadecanoic acid, methyl ester	methyl hexadecanoate	270.5 g/mol	C_17_H_34_O_2_	Ester group (-COO-)	Fatty acid ester	Antimicrobial, Antioxidant, Anti-inflammatory [55]
18.	13.900	0.378	Octanoic acid, methyl ester	methyl octanoate	158.24 g/mol	C_9_H_18_O_2_	Ester group (-COO-)	Fatty acid ester	Antimicrobial Activity, Anticancer Activity [56]
19.	13.900	0.378	Undecanoic acid, 11-bromo-, methyl ester	methyl 11-bromoundecanoate	279.21 g/mol	C_12_H_23_BrO_2_	Ester group (-COO-), Bromo group (-Br)	Bromo fatty acid ester	Antimicrobial Activity, Anti-Biofilm Activity, Antifungal properties [57]
20.	13.900	0.378	Pentadecanoic acid, methyl ester	methyl pentadecanoate	256.42 g/mol	C_16_H_32_O_2_	Ester group (-COO-)	Fatty acid ester	Antimicrobial, Antioxidant, Anti-inflammatory [58]
21.	13.900	0.378	Hexadecanoic acid, methyl ester	methyl hexadecanoate	270.5 g/mol	C_17_H_34_O_2_	Ester group (-COO-)	Fatty acid ester	Antibacterial, Antifungal, Antioxidant, and Hypocholesterolemic effects [59]
22.	14.911	0.264	Ethyl oxamate	ethyl 2-amino-2-oxoacetate	117.1 g/mol	C_4_H_7_NO_3_	Amide group (-CONH₂), Ester group (-COO-)	Amide ester	Potential Anticancer Effects, Antitumor Drug Resistance, Synergistic Effects. [60]
23.	14.911	0.264	L-Alanine, methyl ester	methyl (2S)-2-aminopropanoate	103.12 g/mol	C_4_H_9_NO_2_	Amine group (-NH₂), Ester group (-COO-)	Amino acid ester	Anticancer agents, Anti-HIV activity, Solubility [61]
24.	15.250	0.185	3-Azabicyclo[3.2.2]nonane	3-azabicyclo[3.2.2]nonane	125.21 g/mol	C_8_H_15_N	Amine group (secondary amine, -NH-)	Bicyclic amine	Antibacterial activity, Antiprotozoal Activity, Antimicrobial activity [62]
25.	15.250	0.185	Octodrine	6-methylheptan-2-amine	129.24 g/mol	C_8_H_19_N	Amine group (-NH₂)	Aliphatic amine	Pseudoephedrine, Ephedrine, Antimicrobial properties [63]
26.	15.250	0.185	2-Heptanamine, 5-methyl-	5-methylheptan-2-amine	129.24 g/mol	C_8_H_19_N	Amine group (-NH₂)	Aliphatic amine	Antileishmanial agent, Sympathomimetic Effects, Nasal Decongestant [64]
27.	15.250	0.185	3-azabicyclo 3.2.2 nonane 3-nitroso-	3-nitroso-3-azabicyclo[3.2.2]nonane	154.21 g/mol	C_8_H_14_N_2_O	Nitroso group (-NO) and Amine group (-NH-)	Nitroso derivative of the bicyclic ring	Enzyme Inhibition, Neuroactive Properties, Toxicological Effects [65]
28.	15.250	0.185	Phenylephrine	3-[(1R)-1-hydroxy-2-(methylamino)ethyl]phenol	167.2 g/mol	C_9_H_13_NO_2_	Phenol group (-OH on aromatic ring), Amine group (-NH₂), Hydroxyl group (-OH)	Sympathomimetic amine	Nasal Decongestion, Increased Heart Rate, Vasoconstriction [66]
29.	15.250	0.185	2-Octynoic acid	oct-2-ynoic acid	140.18 g/mol	C_8_H_12_O_2_	Carboxyl group (-COOH) and Alkyne group (-C ≡ C-)	Alkyne carboxylic acid	Antiviral Activity, Optimal reactivity, particularly against Hepatitis C virus (HCV) [67]
30.	15.250	0.185	Glutaraldehyde	pentanedial	100.12 g/mol	C_5_H_8_O_2_	Aldehyde groups (-CHO)	Dialdehyde	Disinfection and Sterilization, Tissue Fixation, Biomaterial Modification [68]
31.	15.250	0.185	Cystine	(2R)-2-amino-3-[[(2R)-2-amino-2-carboxyethyl]disulfanyl]propanoic acid	240.3 g/mol	C_6_H_12_N_2_O_4_S_2_	Thiol group (-SH), Carboxyl group (-COOH) and Amine group (-NH₂)	Amino acid (disulfide-linked)	Protein stabilization, antioxidant defense, Anti-aging Effects [69]
32.	15.680	0.120	11,14,17-Eicosatrienoic acid, methyl ester	methyl (11E,14E,17E)-icosa-11,14,17-trienoate	320.5 g/mol	C_21_H_36_O_2_	Ester group (-COO-) and Alkene group (C = C)	Polyunsaturated fatty acid ester	Anti-inflammatory, Antifungal and Antimicrobial activity [70]
33.	15.680	0.120	9,12-Octadecadienoic acid, methyl ester, (E,E)	methyl (9Z,12Z)-octadeca-9,12-dienoate	294.5 g/mol	C_19_H_34_O_2_	Ester group (-COO-) and Alkene group (C = C)	Polyunsaturated fatty acid ester	Antioxidant, Anti-cancer, and Anti-inflammatory, Antibacterial and Antifungal [71]
34.	15.680	0.120	2-Decyn-1-ol	dec-2-yn-1-ol	154.25 g/mol	C_10_H_18_O	Hydroxyl group (-OH) and Alkyne group (-C ≡ C-)	Alkenyl alcohol	Antimicrobial Properties, Antioxidant Activity, Cytotoxicity or Anticancer Activity [72]
35.	15.680	0.120	10-Undecyn-1-ol	undec-10-yn-1-ol	168.28 g/mol	C_11_H_20_O	Hydroxyl group (-OH) and Alkyne group (-C ≡ C-)	Alkenyl alcohol	Antifungal, Antimicrobial, Antioxidant [73]
36.	15.680	0.120	7-Oxabicyclo[4.1.0]heptane, 3-oxiranyl-	3-(oxiran-2-yl)-7-oxabicyclo[4.1.0]heptane	140.18 g/mol	C_8_H_12_O_2_	Ether group (C-O-C) and Epoxide group (three-membered ring with O)	Bicyclic ether	Antimalarial and Antimicrobial activities, antimicrobial activity [74]
37.	15.680	0.120	5,10-Dioxatricyclo[7.1.0.0(4,6)]decane	5,10-dioxatricyclo[7.1.0.04,6]decane	140.18 g/mol	C_8_H_12_O_2_	Ether group (C-O-C)	Polycyclic ether	Antimalarial and Antimicrobial activities, antimicrobial activity [75]
38.	15.680	0.120	9-Dodecyn-1-ol	dodec-9-yn-1-ol	182.3 g/mol	C_12_H_22_O	Hydroxyl group (-OH) and Alkyne group (-C ≡ C-)	Alkenyl alcohol	Pest Control Applications, Sex Pheromone, Attraction to Males [76]
39.	15.680	0.120	2-Nonyn-1-ol	non-2-yn-1-ol	140.22 g/mol	C_9_H_16_O	Hydroxyl group (-OH) and Alkyne group (-C ≡ C-)	Alkenyl alcohol	Antituberculosis activity as well as Antifungal activity [77]
40.	15.680	0.120	2-Octyn-1-ol	oct-2-yn-1-ol	126.2 g/mol	C_8_H_14_O	Hydroxyl group (-OH) and Alkyne group (-C ≡ C-)	Alkenyl alcohol	Antifungal, Antimicrobial, Antioxidant [78]
41.	15.680	0.120	2-Heptanamine, 5-methyl-	5-methylheptan-2-amine	129.24 g/mol	C_8_H_19_N	Amine group (-NH₂)	Primary amine	anti-leishmanial activity, Cardiovascular Agents [79]
42.	15.680	0.120	Butanal, 3-methyl-	3-methylbutanal	86.13 g/mol	C_5_H_10_O	Aldehyde group (-CHO)	Aldehyde	Antimalarial and Antimicrobial activities, antimicrobial activity [80]
43.	15.680	0.120	Urea, butyl-	butylurea	116.16 g/mol	C_5_H_12_N_2_O	Amide group (-CONH₂)	Substituted urea derivative	antimicrobial activity, particularly against certain bacteria and fungi [81]
44.	15.680	0.120	1,3-Dioxolane, 4-methyl-	4-methyl-1,3-dioxolane	88.11 g/mol	C_4_H_8_O_2_	1,3-dioxolane ring	Dioxolane	antibacterial and antifungal properties [82,83][83]
45.	15.680	0.120	3,3’-Iminobispropylamine	N’-(3-aminopropyl)propane-1,3-diamine	131.22 g/mol	C_6_H_17_N_3_	Amine group (-NH₂) and Secondary amine (-NH-)	Diamine	used as a reagent in chemical synthesis, particularly in the creation of certain types of organic compounds [84]
46.	18.856	1.296	1H-Pyrazole-3-carboxylic acid, 4-nitro-	4-nitro-1H-pyrazole-5-carboxylic acid	157.08 g/mol	C_4_H_3_N_3_O_4_	Carboxyl group (-COOH) and Nitro group (-NO₂)	Heterocyclic aromatic compound	Antimicrobial Properties, Antioxidant Activity, Cytotoxicity or Anticancer Activity [85]
47.	18.855	4.341	9-Octadecenamide, (Z)-	(Z)-octadec-9-enamide	281.5 g/mol	C_18_H_35_NO	Amide group (-CONH₂)	Unsaturated fatty acid amide	hypolipidemic, antioxidant, and anti-inflammatory effects [82]
48.	18.855	4.341	13-Docosenamide, (Z)-	(Z)-docos-13-enamide	337.6 g/mol	C_22_H_43_NO	Amide group (-CONH₂)	Unsaturated fatty acid amide	exhibits antimicrobial and anticancer activities [80]
49.	18.855	4.341	(1S,2R,5R)-Hydroxypropan-2-yl)-5-methylcyclohexanol	(1S,2R,5R)-2-(2-hydroxypropan-2-yl)-5-methylcyclohexan-1-ol	172.26 g/mol	C_10_H_20_O_2_	Hydroxyl group (-OH)	Secondary alcohol	Antimicrobial Properties, Antioxidant Activity, Cytotoxicity or Anticancer Activity [86]
50.	18.855	4.341	3-Dodecanol	dodecan-3-ol	186.33 g/mol	C_12_H_26_O	Hydroxyl group (-OH)	Long chain alcohol	antimicrobial and insecticidal effects [87]
51.	20.796	0.233	Tetrahydro-4H-pyran-4-ol	oxan-4-ol	102.13 g/mol	C_5_H_10_O_2_	Hydroxyl group (-OH) and Ether group (C-O-C)	Cyclic alcohol	Tetrahydro-4H-pyran-4-one has potential biological activities, including antimicrobial and insecticidal properties [88]
52.	20.796	0.233	3,6-Dimethylpiperazine-2,5-dione	3,6-dimethylpiperazine-2,5-dione	142.16 g/mol	C_6_H_10_N_2_O_2_	Amide group (-CONH-)	Cyclic diamide	Antimicrobial Properties, Antioxidant Activity, Cytotoxicity or Anticancer Activity [89]
53.	22.955	0.477	Pentanal	pentanal	86.13 g/mol	C_5_H_10_O	Aldehyde group (-CHO)	Aldehyde	potential antifungal effects and interactions with tubulin, a protein involved in cell structure [90]
54.	25.406	0.561	Decanal	decanal	156.26 g/mol	C_10_H_20_O	Aldehyde group (-CHO)	Aldehyde	antifungal, antibacterial, and antioxidant effects [91]
55.	25.406	0.561	1-Octanol, 3,7-dimethyl-	3,7-dimethyloctan-1-ol	158.28 g/mol	C_10_H_22_O	Hydroxyl group (-OH)	Alcohol	antimicrobial, antifungal, and antioxidant properties [92]
56.	25.406	0.561	Acetic acid, 2-ethylhexyl ester	2-ethylhexyl acetate	172.26 g/mol	C_10_H_20_O_2_	Ester group (-COO-)	Ester	Anti-bacterial effect [93]
57.	25.406	0.561	Butanoic acid, 2-methyl-, octyl ester	octyl 2-methylbutanoate	214.34 g/mol	C_13_H_26_O_2_	Ester group (-COO-)	Ester	Antimicrobial activity and shows cytotoxicity against ovarian cancer cell line. [94]
58.	26.808	13.243	3,4-Dimethoxy-dl-phenylalanine	2-amino-3-(3,4-dimethoxyphenyl)propanoic acid	225.24 g/mol	C_11_H_15_NO_4_	Amine group (-NH₂), Carboxylic acid group (-COOH) and Ether group (-O-)	Amino acid derivative	Antioxidant effect against hepatocellular carcinoma [95]
59.	26.808	13.243	Benzeneacetamide, 3,4-dimethoxy-	2-(3,4-dimethoxyphenyl)acetamide	195.21 g/mol	C_10_H_13_NO_3_	Amide group (-CONH₂) and Ether group (-O-)	Amide derivative	Antibacterial activity [96], antioxidant and anti-inflammatory effect [97]
60.	26.808	13.243	3-(3,4-Dimethoxyphenyl)-propionic acid	3-(3,4-dimethoxyphenyl)propanoic acid	210.23 g/mol	C_11_H_14_O_4_	Carboxyl group (-COOH) and Ether group (-O-)	Phenylpropanoic acid derivative	Hepatoprotective property [98] and antioxidant activity [99]
61.	27.140	0.538	Silane, trimethyl[5-methyl-2-(1-methylethyl)phenoxy]-	trimethyl-(5-methyl-2-propan-2-ylphenoxy)silane	222.4 g/mol	C_13_H_22_OSi	Silane group (-Si-) and Ether group (-O-)	Organosilane	Antibacterial and antifungal activities [100]
62.	27.140	0.538	4-tert-Butylphenol, TMS derivative	(4-tert-butylphenoxy)-trimethylsilane	222.4 g/mol	C_13_H_22_OSi	Siloxy group (-OSi(CH₃)₃)	Trimethylsilyl derivative	Antioxidant effect [101], Antibacterial and anti-cancer properties [102]
63.	27.140	0.538	Phloroglucitol	cyclohexane-1,3,5-triol	132.16 g/mol	C_6_H_12_O_3_	Hydroxyl group (-OH)	Polyphenol	Anti-cancer activity against breast cancer [103], antimicrobial activity [104]
64.	27.140	0.538	Silanol, trimethyl-, phosphite (3:1)	tris(trimethylsilyl) phosphite	298.54 g/mol	C_9_H_27_O_3_PSi_3_	Silanol group (-Si-OH) and Phosphite group (-P(OR)₃)	Organosilicon compound	Antimicrobial and antifungal activity [105]
65.	27.140	0.538	1,2-Bis(trimethylsilyl)benzene	trimethyl-lsilylphenyl)silane	222.47 g/mol	C_12_H_22_Si_2_	Siloxy group (-OSi(CH₃)₃)	Aromatic organosilicon compound	Antibacterial and anticancer activities [106]
66.	27.140	0.538	Androsta-3,5-dien-3-ol, 17-acetyl-3-O-(t-butyldimethylsilyl)-	1-[(8S,9S,10R,13S,14S,17S)-3-[tert-butyl(dimethyl)silyl]oxy-10,13-dimethyl-2,7,8,9,11,12,14,15,16,17-decahydro-1H-cyclopenta[a]phenanthren-17-yl]ethanone	428.7 g/mol	C_27_H_44_O_2_Si	Ketone group (C = O) and Siloxy group (-OSiR₃)	Steroid derivative	Antioxidant activity [107]
67.	27.140	0.538	4-Tetradecanol	tetradecan-4-ol	214.39 g/mol	C_14_H_30_O	Hydroxyl group (-OH)	Long chain alcohol	Antibacterial activity [108], anticancer and antioxidant activity [109]
68.	27.751	3.402	Disiloxane, 1,3-diethoxy-1,1,3,3-tetramethyl-	ethoxy-[ethoxy(dimethyl)silyl]oxy-dimethylsilane	222.43 g/mol	C_8_H_22_O_3_Si_2_	Siloxane bond (-Si-O-Si-) and Ether group (-O-)	Siloxane compound	Not widely researched for its bioactivity, but it is useful reagent in organic chemistry [110]
69.	27.751	3.402	Acetamide, y)-3-methoxyphenyl]ethyl]-	[4-(2-acetamido-1-acetyloxyethyl)-2-methoxyphenyl] acetate	309.31 g/mol	C_15_H_19_NO_6_	Amide group (-CONH-), Ester group (-COO-), and Ether group (-O-)	Acetamide derivative	Antioxidant and anti-inflammatory activities [111]
70.	28.954	0.471	Androsta-3,5-dien-3-ol, 17-acetyl-3-O-(t-butyldimethylsilyl)-	1-[(8S,9S,10R,13S,14S,17S)-3-[tert-butyl(dimethyl)silyl]oxy-10,13-dimethyl-2,7,8,9,11,12,14,15,16,17-decahydro-1H-cyclopenta[a]phenanthren-17-yl]ethanone	428.7 g/mol	C_27_H_44_O_2_Si	Ketone group (C = O) and Siloxy group (-OSiR₃)	Steroid derivative	Antioxidant activity [107]
71.	28.954	0.471	Pentasiloxane, dodecamethyl-	bis[[dimethyl(trimethylsily-loxy)silyl]oxy]-dimethylsilane	384.84 g/mol	C_12_H_36_O_4_Si_5_	Siloxane bond (-Si-O-Si-)	Siloxane polymer	Antifungal, antioxidant and antimicrobial activity [112]
72.	28.837	4.695	Benzeneethanamine, 2,5-dimethoxy-.alpha.,4-dimethyl-	1-(2,5-dimethoxy-4-methylphenyl)propan-2-amine	209.28 g/mol	C_12_H_19_NO_2_	Amine group (-NH₂) and Ether groups (-O-)	Aromatic amine derivative	Anti-obesity activity [113]
73.	28.837	4.695	5H-Benzo[b]pyran-8-ol, 2,3,5,5,8a-pentamethyl-6,7,8,8a-tetrahydro-	2,3,5,5,8a-pentamethyl-7,8-dihydro-6H-chromen-8-ol	222.32 g/mol	C_14_H_22_O_2_	Hydroxyl group (-OH) and Benzopyran ring system	Polycyclic alcohol	Antimicrobial activity [114]
74.	28.837	4.695	4’,4’‘-Dinitrodibenzo[b,k]-1,4,7,10,13,16-hexaoxacyclooctadecan-2,11-diene	11,25-dinitro-2,5,8,15,18,21-hexaoxatricyclo[20.4.0.09,14]hexacosa-1(22),9(14),10,12,23,25-hexaene	450.4 g/mol	C_20_H_22_N_2_O_10_	Nitro groups (-NO₂) and Ether groups (-O-)	Polyether macrocycle	Antitumor activity [115]
75.	32.107	1.365	Methyltris(trimethylsiloxy)silane	trimethyl-[methyl-bis(trimethylsilyloxy)silyl]oxysilane	310.68 g/mol	C_10_H_30_O_3_Si_4_	Siloxy group (-OSi(CH₃)₃)	Silane derivative	Antibacterial activity [116], antioxidant effect [117]

**Fig 2:**
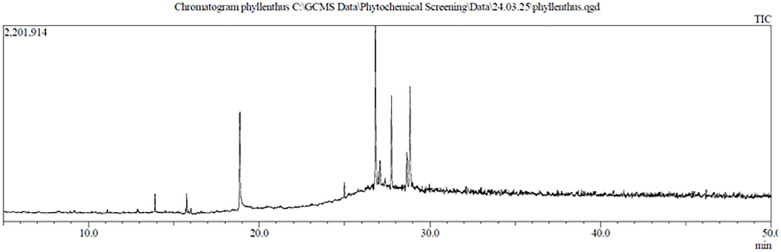
Chromatogram of GC-MS analysis of *Phyllanthus niruri* leaf extract.

GC-MS profiling provided a semi-quantitative overview of the phytochemical constituents of *Phyllanthus niruri*, where compound abundance was expressed as relative peak area percentages. The compounds in Table 3 have concentrations ranging from 0.120 µg/mL to 13.243 µg/mL, indicating a wide range of chemical abundance. Among the most abundant compounds are 3,4-Dimethoxy-dl-phenylalanine, Benzeneacetamide, 3,4-dimethoxy-, and 3-(3,4-Dimethoxyphenyl)-propionic acid, each present at 13.243 µg/mL. Moderately abundant compounds include Benzeneethanamine, 2,5-dimethoxy-. alpha.,4-dimethyl- and 5H-Benzo[b]pyran-8-ol, 2,3,5,5,8a-pentamethyl-6,7,8,8a-tetrahydro-, with concentrations of 4.695 µg/mL, and Disiloxane, 1,3-diethoxy-1,1,3,3-tetramethyl- at 3.402 µg/mL. Several compounds such as 2-Octyn-1-ol, 2-Heptanamine, 5-methyl-, and Butanal, 3-methyl- appear in lower amounts, with concentrations near 0.120 µg/mL (Fig 2).

The extract includes a diverse group of chemicals such as amino acids, amines, fatty acid esters, alcohols, and aromatic derivatives, many of which exhibit bioactive properties including antioxidant, antimicrobial, anti-inflammatory, and anticancer effects. Notably, compounds with moderate retention times often show higher concentrations, suggesting they are more readily separated and quantified using this analytical method. Meanwhile, compounds with lower concentrations are frequently those with longer retention times, possibly due to complex interactions with the column.

In addition to retention time, concentration data play a vital role in quantifying the amounts of various compounds present in a sample. This quantitative information enhances compound identification and confirmation, which is essential in disciplines such as pharmacology, toxicology, biochemical analysis, and environmental testing. In industrial applications, particularly within the pharmaceutical and chemical manufacturing sectors, accurate monitoring of plant-derived chemical concentrations is essential for ensuring quality control and meeting regulatory standards. In this study, various compounds were quantitatively identified in the methanolic extract of *Phyllanthus niruri*. By analyzing both retention times and concentration values, a comprehensive chemical profile was established. This approach not only aids in the precise identification of individual bioactive constituents but also enables accurate quantification, which is crucial for consistency in formulation and efficacy in therapeutic applications.

The relative abundance of dimethoxy derivatives and long-chain fatty acid esters indicates that these compounds may act synergistically in antibacterial mechanisms. When compared with earlier Phyllanthus species studies, three compounds Benzeneacetamide (3,4-dimethoxy-), 3-(3,4-Dimethoxyphenyl)-propionic acid, and 5H-Benzo[b]pyran-8-ol—appear as newly reported constituents in *Phyllanthus niruri* leaf, highlighting the novelty of this GC–MS profile.

### 4.1. Chemical classification of the identified compounds

**Fig 3:**
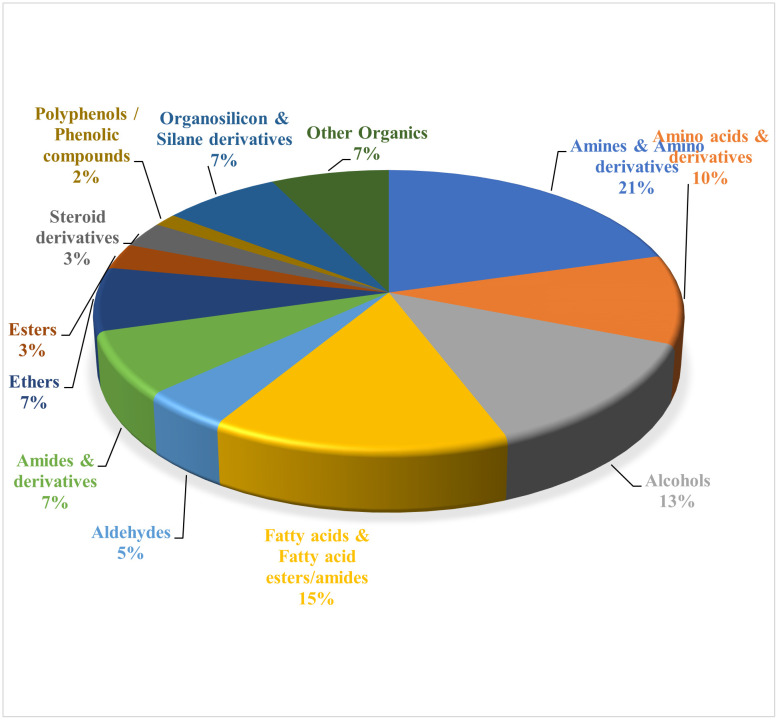
Percentage composition of identified chemical classes.

The compounds listed in Table 3 exhibit a diverse chemical profile comprising amines and amino derivatives (21%) (Aliphatic, Aromatic, Cyclic, Bicyclic, Diamine, Phenethylamine, Amino alcohol); amino acids and derivatives (10%) (including cystine, alanine derivatives); alcohols (13%) (including Alkenyl, Cycloalcohol, Polycyclic, Long chain alcohols); fatty acids and esters (15%) (saturated, unsaturated, polyunsaturated, bromo derivatives); aldehydes (5%); amides and derivatives (7%) (including urea, cyclic diamide); ethers and polyethers (7%) (Bicyclic ether, Polycyclic ether, Polyether macrocycle, Siloxane polymer, Disiloxane); esters (3%) (excluding fatty acid esters); steroid derivatives (3%); polyphenols (~2%); organosilicon & silane derivatives (7%); and other organic molecules (7%) (Fig 3). There are reports that amines and amino derivatives play crucial roles as neurotransmitters and exhibit diverse pharmacological properties including antimicrobial and sympathomimetic effects. Amino acids and their derivatives are essential for protein biosynthesis, metabolic regulation, and muscle maintenance. Fatty acids and esters contribute to membrane structure and possess antioxidant, anti-inflammatory, and anticancer properties. Alcohols are often associated with antimicrobial and antioxidant activities. Organosilicon compounds and ethers have been explored for their biocompatibility and bioavailability enhancement. The presence of steroid derivatives enriches the extract’s potential for hormonal modulation and anticancer effects. Polyphenolic compounds are well known for their antioxidant and cardioprotective activities [118].

### 4.2. Distribution of identified compounds based on major biological activities

**Fig 4:**
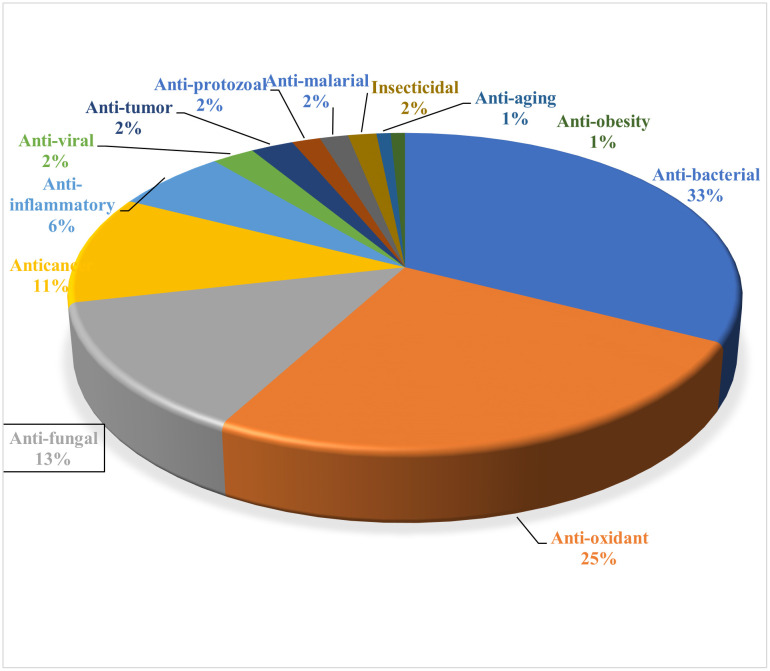
Distribution of identified compounds according to major biological activities (%).

*Phyllanthus niruri*, commonly known as “stone breaker,” is a medicinal herb with a wide range of pharmacological activities supported by both traditional use and scientific research. It exhibits potent antiviral effects, particularly against Hepatitis B virus, by inhibiting viral DNA polymerase. Its hepatoprotective properties help regenerate liver cells and protect against toxins, making it valuable in treating cirrhosis and hepatitis. Rich in flavonoids and polyphenols, the plant also acts as a strong antioxidant, reducing oxidative stress and cellular damage. Anti-inflammatory compounds in plant leaf suppress cytokines and enzymes like COX-2, while its anticancer activity involves inducing apoptosis and inhibiting tumor growth. It shows antidiabetic effects by improving insulin sensitivity and lowering blood glucose levels, and its antiplasmodial action targets malaria parasites. Traditionally used for kidney health, *Phyllanthus niruri* demonstrates nephroprotective and antilithic effects by preventing and dissolving kidney stones (Fig 4). Additionally, it has antimicrobial activity against pathogens like *Staphylococcus aureus* and *Candida albicans*. These effects are attributed to bioactive compounds such as *phyllanthin, hypophyllanthin*, quercetin, rutin, alkaloids, tannins, and saponins [15], confirm its therapeutic potential across multiple biological pathways.

## 5. *In-vitro* antibacterial activity analysis

The antibacterial potential of five different organic solvent fractions derived from the leaf of *Phyllanthus* was evaluated against a panel of nine pathogenic bacterial strains, consisting of both Gram-positive and Gram-negative species. The antimicrobial results revealed that leaf extract demonstrated notable inhibitory effects against these pathogens. This suggests that the plant contains bioactive compounds with promising antibacterial properties. The findings support the potential of *Phyllanthus niruri* as a natural source for developing plant-based antimicrobial agents and align with the growing interest in phytochemicals as alternatives or complements to conventional treatments [52].

Among the tested fractions, the ESF exhibited the most prominent antibacterial effect, particularly against *Escherichia coli* (17.2 mm), *Shigella dysenteriae* (17.4 mm), and *Bacillus subtilis* (15.1 mm). The MSF also showed strong inhibition, notably against *Staphylococcus aureus* (20.5 mm) and *E. coli* (14.1 mm). Other fractions, including PSF, CTF, and CSF displayed moderate to weak antibacterial activity, while some bacteria showed no response to certain extracts. The standard antibiotic Che-5 demonstrated the highest inhibition zones (26–33 mm) across all test organisms, confirming its potent antibacterial effect all are presented in Table 4. Overall, the results indicate that the ESF and MSF fractions possess significant antibacterial properties, suggesting the presence of bioactive compounds with broad-spectrum antimicrobial potential.

**Table 4:** Antibacterial activity by different extractives of leaf of *Phyllanthus niruri* through agar diffusion method.

Test bacteria	Inhibitory zone diameter (mm)					
	MSF	PSF	CTF	CSF	ESF	Che-5
**Gram positive bacteria**						
*Bacillus subtilis*	10.50 ± 0.21^b^	–	3.51 ± 0.50^c^	–	15.10 ± 0.52^b^	33.20 ± 0.96^c^
*Sarcinalutea*	4.31 ± 0.42^c^	7.21 ± 0.55^b^	11.51 ± 0.57^a^	9.21 ± 0.75a^b^	12.11 ± 0.45^a^	30.40 ± 0.72^c^
*Staphylococcus* *aureus*	20.50 ± 0.25^a^	11.40 ± 0.31^b^	9.52 ± 0.15^bc^	8.51 ± 0.45^c^	14.11 ± 0.10^c^	28.03 ± 0.15^d^
*Bacillus cereus*	9.20 ± 0.25^c^	11.10 ± 0.15^b^	9.02 ± 0.32^c^	9.10 ± 0.15^c^	13.20 ± 0.81^c^	26.01 ± 0.31^d^
**Gram negative bacteria**						
*E. coli*	14.11 ± 0.15^b^	7.51 ± 0.15^c^	9.32 ± 0.52^c^	9.20 ± 0.21^c^	17.20 ± 0.52^a^	27.02 ± 0.35^d^
*Shigella dysenteriae*	11.51 ± 0.21^b^	–	–	6.40 ± 0.87^c^	17.40 ± 0.95^a^	30.01 ± 0.15^d^
*Vibrio mimicus*	12.51 ± 0.21^b^	11.10 ± 0.25^b^	–	–	16.01 ± 0.95^a^	30.01 + 0.92^c^
*parahemolyticus*	8.50 ± 0.12^a^	9.20 ± 0.20^c^	8.20 ± 0.35^c^	–	13.21 ± 0.86^b^	27.02 + 0.91^a^
*Salmonella typhi*	9.30 ± 0.17^c^	8.81 ± 0.18^c^	9.10 ± 0.85^c^	–	14.40 ± 0.64^b^	30.02 + 0.05^a^

Values represent mean ± standard deviation (n = 3). Means within a row followed by the same superscript letter (a–d) do not differ significantly (p < 0.05; Duncan’s Multiple Range Test, DMRT). Che-5: Ciprofloxacin (5 µg/disc, standard). (–): No inhibition observed. MSF: Methanol-soluble fraction; PSF: Petroleum ether-soluble fraction; CTF: Chloroform-soluble fraction; CSF: Carbon tetrachloride-soluble fraction; ESF: Ethyl acetate-soluble fraction.

*Phyllanthus niruri* exhibited notable antibacterial activity against nine clinically significant bacterial strains, including four Gram-positive and five Gram-negative species selected for in *vitro* evaluation. These bacterial strains are responsible for various human infections presented in Table 5, including systemic, gastrointestinal, respiratory, and dermal diseases. Testing against this diverse group allowed for a comprehensive assessment of the broad-spectrum antimicrobial potential of leaf extracts*.*

**Table 5:** List of selected gram-positive and gram-negative bacteria selected for *in-vitro* analysis.

Name of the Bacteria	Disorders caused by the experimental organism
**Gram positive bacteria:**	
*Bacillus subtilis*	Bacteraemia, endocarditis, pneumonia, and septicaemia.
*Bacillus cereus*	Emetic (vomiting) syndrome and diarrheal syndrome.
*Staphylococcus aureus*	Abscesses (boils), furuncles, and cellulitis.
*Sarcina lutea*	Ventriculitis, peritonitis, endophthalmitis, keratolysis and septic arthritis.
**Gram negative bacteria:**	
*Salmonella typhi*	Typhoid fever or paratyphoid fever.
*Vibrio parahaemolyticus*.	Gastroenteritis (gastro).
*Escherichia coli*	Meningitis, abdominal and pelvic infection, pneumonia, and urinary tract infection.
*Vibrio mimicus*	Gastroenteritis in humans.
*Bacillus parahaemolyticus.*	Diarrhoea, abdominal cramps, and nausea.

The selected Gram-positive and Gram-negative bacteria are clinically significant pathogens responsible for various human infections. They were included to assess the antibacterial potential of *Phyllanthus niruri* extracts through in-vitro analysis.

The MIC values of *Phyllanthus niruri* fractions against selected bacteria are presented in Table 6. Among Gram-positive strains, the ESF exhibited the strongest inhibitory effects, with MICs ranging from 7.8–15.6 mg/mL, followed by moderate activity from MSF and PSF, CTF and CSF showed comparatively weaker inhibition. For Gram-negative bacteria, ESF again demonstrated the most pronounced activity (7.8–19.5 mg/mL), while other fractions showed variable or minimal inhibition. The standard antibiotic Ciprofloxacin (Che-5) displayed the lowest MIC values (0.25–1.0 µg/mL), significantly more effective than the plant-derived fractions (p < 0.05), presented in Table 6.

**Table 6:** Minimum inhibitory concentration (MIC) of *Phyllanthus niruri* leaf fractions against tested bacteria.

Test bacteria	MSF (mg/mL)	PSF (mg/mL)	CTF (mg/mL)	CSF (mg/mL)	ESF (mg/mL)	Che-5 (µg/mL)
**Gram-positive bacteria**						
*Bacillus subtilis*	15.60 ± 1.20ᵇ	–	25.40 ± 1.50ᶜ	–	10.20 ± 0.80ᵃ	0.50 ± 0.02ᵈ
*Sarcina lutea*	20.10 ± 1.40ᶜ	12.50 ± 0.9ᵇ	8.31 ± 0.71ᵃ	14.20 ± 1.10ᵇ	6.50 ± 0.51ᵃ	0.50 ± 0.03ᵈ
*Staphylococcus aureus*	8.71 ± 0.61ᵃ	14.01 ± 1.21ᵇ	18.20 ± 1.01ᶜ	16.50 ± 0.91ᵇᶜ	5.40 ± 0.41ᵃ	0.25 ± 0.01ᵈ
*Bacillus cereus*	12.31 ± 0.81ᵇ	9.51 ± 0.70ᵃ	11.01 ± 0.91ᵇ	10.81 ± 0.80ᵇ	5.81 ± 0.41ᵃ	0.50 ± 0.02ᶜ
**Gram-negative bacteria**						
*Escherichia coli*	14.21 ± 1.01ᵇ	18.01 ± 1.21ᶜ	12.50 ± 0.90ᵇ	16.80 ± 1.10ᶜ	8.90 ± 0.60ᵃ	0.50 ± 0.02ᵈ
*Shigella dysenteriae*	19.50 ± 1.31ᶜ	–	–	17.20 ± 1.11ᵇ	7.80 ± 0.50ᵃ	0.25 ± 0.01ᵈ
*Vibrio mimicus*	12.80 ± 0.91ᵇ	11.51 ± 0.80ᵃ	–	–	8.20 ± 0.60ᵃ	0.25 ± 0.01ᶜ
*Vibrio parahaemolyticus*	18.50 ± 1.19ᶜ	13.20 ± 0.90ᵇ	17.01 ± 1.01ᶜ	–	11.31 ± 0.71ᵃ	0.50 ± 0.02ᵈ
*Salmonella typhi*	13.51 ± 0.90ᵇ	16.80 ± 1.21ᶜ	12.71 ± 1.02ᵇ	–	9.51 ± 0.60ᵃ	0.25 ± 0.01ᵈ

Values are presented as mean ± SD (n = 3). “–” indicates no activity at tested concentrations.

Superscript letters (a, b, c, d) indicate statistically significant differences among fractions and Che-5 within the same row (*p* < 0.05). Statistical analysis: one-way ANOVA followed by Duncan’s Multiple Range Test.

The MBC values of *Phyllanthus niruri* fractions against nine bacterial strains are summarized in Table 7. Among Gram-positive bacteria, the ESF showed the strongest activity, with MBCs of 10.8–20.4 mg/mL, followed by moderate effects from the MSF and PSF. The CTF and CSF showed comparatively weaker activity. For Gram-negative bacteria, ESF again exhibited the lowest MBC values (15.6–22.6 mg/mL), indicating broad-spectrum bactericidal potential. Other fractions displayed moderate to low activity, with some showing no bactericidal effect. The reference drug Ciprofloxacin (Che-5) showed the highest potency (0.5–1.0 µg/mL), significantly more active than all extract fractions (*p* < 0.05), presented in Table 7.

**Table 7:** Minimum bactericidal concentration (MBC) of *Phyllanthus niruri* leaf fractions against tested bacteria.

Test bacteria	MSF (mg/mL)	PSF (mg/mL)	CTF (mg/mL)	CSF (mg/mL)	ESF (mg/mL)	Che-5 (µg/mL)
**Gram-positive bacteria**						
*Bacillus subtilis*	31.20 ± 1.50^b^	–	50.80 ± 2.01^c^	–	20.41 ± 1.01^a^	1.01 ± 0.03^d^
*Sarcina lutea*	40.20 ± 1.71^c^	25.01 ± 1.21^b^	16.61 ± 0.81^a^	28.40 ± 1.30^b^	13.01 ± 0.70^a^	1.01 ± 0.04^d^
*Staphylococcus aureus*	17.41 ± 0.81^a^	28.01 ± 1.50^b^	36.40 ± 1.80^c^	33.01 ± 1.71^bc^	10.81 ± 0.51^a^	0.51 ± 0.02^d^
*Bacillus cereus*	24.61 ± 1.01^b^	19.01 ± 0.90^a^	22.01 ± 1.01^b^	21.60 ± 1.01^b^	11.61 ± 0.51^a^	1.01 ± 0.03^c^
**Gram-negative bacteria**						
*sEscherichia coli*	28.40 ± 1.20^b^	36.01 ± 1.50^c^	25.01 ± 1.21^b^	33.60 ± 1.41^c^	17.81 ± 0.80 ^a^	1.01 ± 0.03^d^
*Shigella dysenteriae*	39.01 ± 1.61^c^	–	–	34.40 ± 1.40^b^	15.61 ± 0.70 ^a^	0.51 ± 0.02^d^
*Vibrio mimicus*	25.61 ± 1.20^b^	23.01 ± 1.01^a^	–	–	16.41 ± 0.80 ^a^	0.51 ± 0.02^c^
*Vibrio parahaemolyticus*	37.01 ± 1.51^c^	26.41 ± 1.20^b^	34.01 ± 1.50 ^c^	–	22.61 ± 1.01^a^	1.011 ± 0.03^d^
*Salmonella typhi*	27.01 ± 1.20^b^	33.60 ± 1.50^c^	25.41 ± 1.20 ^b^	–	19.01 ± 0.81^a^	0.51 ± 0.02^d^

MBC = Minimum Bactericidal Concentration; lowest concentration of extract (mg/mL) or Ciprofloxacin (µg/mL) that kills ≥99.9% of bacteria. Values are mean ± SD (n = 3). “–” indicates no bactericidal activity at tested concentrations. Superscript letters (a, b, c, d) indicate statistically significant differences among fractions and Che-5 within the same row. Statistical analysis: one-way ANOVA followed by Duncan’s Multiple Range Test, *p* < 0.05.

The antibacterial activity was assessed using the disc diffusion method, where 1000.0 μg/disc of each extract was tested for its ability to inhibit microbial growth. The zone of inhibition produced by each fraction was measured in millimetres and compared with the standard antibiotic, ciprofloxacin (5.0 μg/disc), known for its broad-spectrum antibacterial action through inhibition of bacterial DNA gyrase and topoisomerase. Ciprofloxacin binds to bacterial DNA gyrase with 100 times the affinity of mammalian DNA gyrase [19]. The diameter of the inhibition zones for each bacterial strain is presented in Table 4.

The study investigated the antibacterial activity of various solvent fractions derived from the medicinal plant *Phyllanthus niruri* against a range of pathogenic bacteria. The results clearly indicate that the efficacy of these extracts depends heavily on the polarity of the solvents used. MSF, being highly polar, the ESF, being moderately polar, demonstrated the strongest antibacterial activity. The zone of inhibition for MSF ranged from 4.3 mm to 20.5 mm, while for ESF it ranged from 12.1 mm to 17.4 mm. Notably, these extracts were effective against both Gram-positive and Gram-negative bacteria, with *Staphylococcus aureus* showing a zone of 20.5 mm (MSF) and 14.1 mm (ESF), and *Escherichia coli* showing 14.1 mm (MSF) and 17.2 mm (ESF). This indicates a strong sensitivity of these pathogens to polar compounds in *Phyllanthus niruri*.

In contrast, extracts obtained using non-polar solvents namely the PSF, CSF, and CTF exhibited minimal to no antibacterial activity, with inhibition zones generally in the range of 3.5 mm to 11.5 mm, or absent altogether. This limited activity is likely because non-polar solvents do not efficiently extract key bioactive compounds such as phenolics, flavonoids, and glycosides, which are typically polar and known for their antimicrobial properties.

The standard antibiotic control (Che-5) showed the highest inhibitory zones, ranging from 26.0 mm to 33.2 mm, serving as a benchmark for evaluating the plant extract’s effectiveness. Overall, the study suggests that polar fractions of leaf are more potent in inhibiting the growth of pathogenic bacteria. These findings support the traditional medicinal use of leaf extract and highlight its potential as a source of natural antibacterial agents, particularly against pathogens such as *S. aureus* and *E. coli.*

These findings conclude that *Phyllanthus niruri* serve as a promising natural reservoir of antibacterial compounds, reinforcing its potential in the development of plant-based therapeutic agents for combating infectious diseases [54]. In this study, the antibacterial effects observed across all nine bacterial strains confirm that the extracts contain active compounds with the ability to fight infections. This supports the traditional use of this plant for treating bacterial illnesses.

The antibacterial activity of leaf fractions was assessed by MIC and MBC against Gram-positive and Gram-negative bacteria. Among all fractions, the ESF showed the strongest inhibition, with MICs of 7.8–19.5 mg/mL and corresponding MBCs of 10.8–22.6 mg/mL, indicating potent bacteriostatic and bactericidal effects. MSF and PSF exhibited moderate activity (MIC 8.7–20.1 mg/mL, MBC 17.4–40.2 mg/mL), while CTF and CSF were weaker. Gram-negative bacteria were generally less sensitive due to the outer membrane barrier, though ESF still showed significant inhibition [119]. Ciprofloxacin (Che-5) was the most effective, with MICs and MBCs of 0.25–1.0 µg/mL (p < 0.05). Antibacterial activity is likely due to semi-polar phytochemicals such as flavonoids, tannins, and phenolics, which disrupt cell walls and inhibit enzymes [120]. These findings support the traditional use of *Phyllanthus. niruri* and its potential for natural antimicrobial development.

Comparable MIC and MBC results have been previously reported for medicinal plants with phenolic and flavonoid-rich fractions, where enhanced membrane permeability and protein denaturation contribute to bacterial cell death [26]. Thus, the low MIC and MBC values observed for leaf extracts substantiate their strong antibacterial potential and support the presence of bioactive phytochemicals acting through multiple mechanisms of action.

This study examined the leaf of *Phyllanthus niruri* and identified several natural compounds, including flavonoids, tannins, and saponins. These compounds showed inhibiting the growth of harmful bacteria [59].

### 5.1. *In-silico* study result

**Fig 5:**
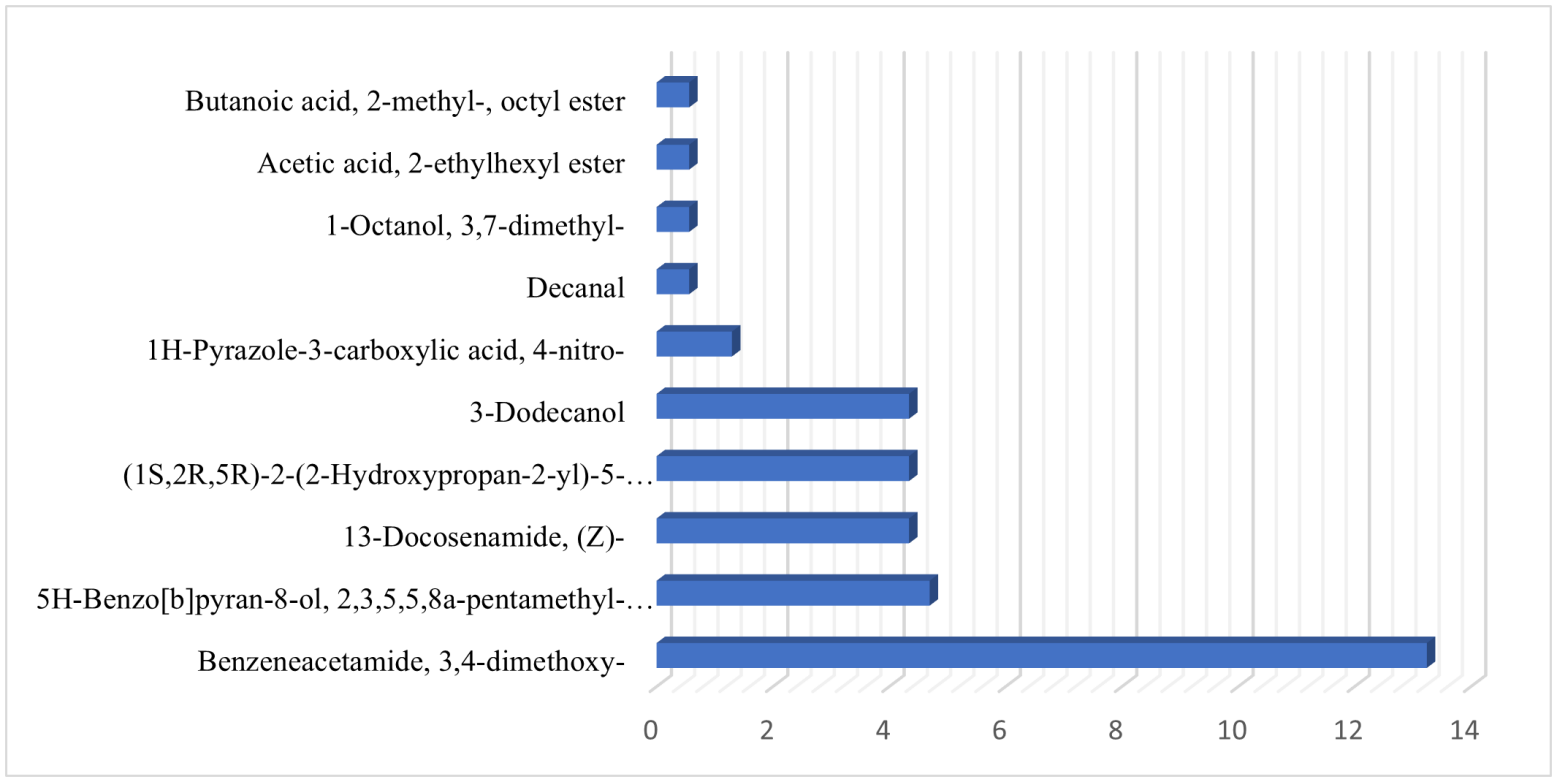
Abundance of chosen compounds.

To support and extend the observed in vitro antimicrobial activity of leaf extract, a comprehensive in silico approach was undertaken. Among the 75 compounds identified via GC-MS analysis, 10 major phytoconstituents were selected for molecular docking studies based on their high concentration per peak area and well-documented antimicrobial properties to further assess their antimicrobial potential against both gram-positive and gram-negative bacterial strains. These compounds were hypothesized to play key roles in the observed biological effects. This *in silico* study was conducted to identify the key bioactive compounds potentially responsible for the antimicrobial activity of leaf extract at the molecular level, aiming to link experimental findings with computational predictions. Among the ten major compounds detected, Benzeneacetamide (3,4-dimethoxy-) emerged as the most abundant, demonstrating notable antimicrobial and anti-inflammatory properties. Additionally, 5H**-**Benzo[b]pyran-8-ol and 13-Docosenamide also exhibited significant pharmacological potential, including antioxidant, anticancer, and antibacterial activities [121]. Their abundance is illustrated in Fig 5, while the selection criteria and properties are detailed in Table 8. The high concentration and favorable bioactivity profiles of these compounds highlight their potential as promising candidates for future drug development.

**Table 8:** Selection of 10 compounds with significant antimicrobial effect based on the highest concentration per volume of extract.

Serial No.	Conc. (ug/ml)	PubChem ID	Compound name
1	13.243	79746	Benzeneacetamide, 3,4-dimethoxy-
2	4.695	540443	5H-Benzo[b]pyran-8-ol, 2,3,5,5,8a-pentamethyl-6,7,8,8a-tetrahydro-
3	4.341	5365371	13-Docosenamide, (Z)-
4	4.341	9920491	(1S,2R,5R)-2-(2-Hydroxypropan-2-yl)-5-methylcyclohexanol
5	4.341	139108	3-Dodecanol
6	1.296	219739	1H-Pyrazole-3-carboxylic acid, 4-nitro-
7	0.561	8175	Decanal
8	0.561	7792	1-Octanol, 3,7-dimethyl-
9	0.561	7635	Acetic acid, 2-ethylhexyl ester
10	0.561	520455	Butanoic acid, 2-methyl-, octyl ester

**Fig 6:**
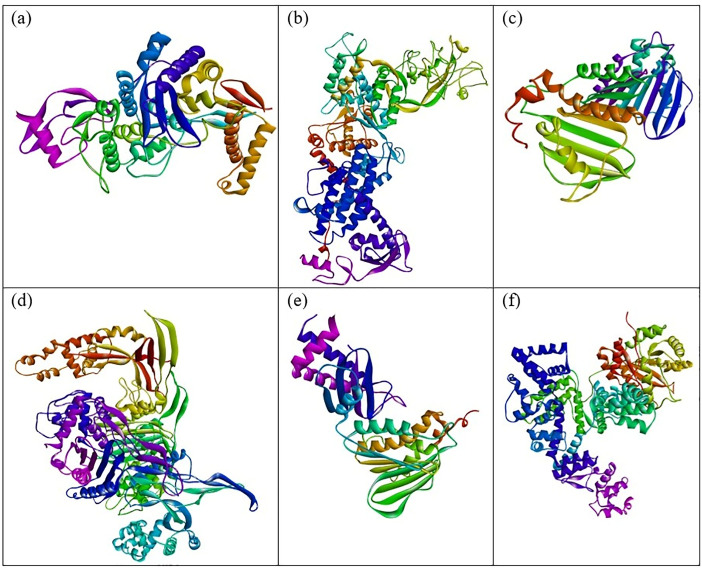
Three-dimensional structures of selected target proteins from *Staphylococcus aureus.* **(a)** Penicillin-Binding Protein 1B (PBP1B), **(b)** Isoleucyl-tRNA Synthetase (IleRS), **(c)** DNA Gyrase Subunit B (GyrB), and **(d)** Penicillin-Binding Protein 3 (PBP3); and from *Escherichia coli* **(e)** DNA Gyrase Subunit B (GyrB) and **(f)** Catalyticα-subunit of DNA Polymerase III (Pol III α-subunit).

To further investigate the antimicrobial efficacy of *Phyllanthus niruri* extract at the molecular level, the selected ligands were subjected to molecular docking analysis against key protein targets associated with *Staphylococcus aureus* and *Escherichia coli*. These bacterial strains were previously identified as highly susceptible to the extract during *in vitro* screening, making them suitable models for correlating computational findings with experimental results. The 3D structures of the selected target proteins are presented in Fig 6, providing a visual reference for the molecular docking analysis. For *Staphylococcus aureus*, four targets were chosen, which includes Penicillin-Binding Protein 1B (PBP1B) [PDB ID: 2Y2I], Isoleucyl-tRNA synthetase (IleRS) [PDB ID: 1FFY], DNA Gyrase Subunit B (GyrB) [PDB ID: 3G75], and Penicillin-Binding Protein 3 (PBP3) [PDB ID: 3VSL]; while for *Escherichia coli*, two critical targets were selected, including DNA Gyrase Subunit B (GyrB) [PDB ID: 4PRX] and the catalytic α-subunit of DNA Polymerase III (Pol III α-subunit) [PDB ID: 2HNH].

To establish a comparative benchmark, ciprofloxacin, a well-established broad-spectrum antibiotic, was used as the standard drug. Molecular docking simulations were performed between each selected ligand and the respective target proteins, as well as between ciprofloxacin and the same protein targets. Before the docking procedure, grid box dimensions (X, Y, and Z coordinates) were defined for each target protein to ensure accurate ligand-binding predictions. These docking parameters are summarized in Table 9. The results of the molecular docking analysis, including binding affinities of the selected phytocompounds and ciprofloxacin against the six protein targets. These findings provide insight into the potential molecular interactions driving the antimicrobial activity of *Phyllanthus niruri* and highlight specific compounds that may play a major role in inhibiting vital bacterial proteins.

**Table 9:** Docking grid box dimensions for protein ligand complexes.

Docking	Grid box size (Dimensions)		
	X	Y	Z
2Y2I with all ligands	55.0086	81.7345	63.7206
1FFY with all ligands	80.3354	101.6714	120.3884
3G75 with all ligands	72.9139	47.1325	70.6265
3VSL with all ligands	78.4669	122.2894	84.3200
4PRX with all ligands	59.4163	72.6365	54.4502
2HNH with all ligands	91.7009	95.2282	83.1877

The docking parameters listed in Table 9 show the grid box dimensions used for each protein-ligand docking simulation. These grid boxes define the 3D space where the ligand binds to the target protein, and their sizes vary depending on the structural characteristics of each protein. The protein 2Y2I used a moderate grid size (X = 55.01, Y = 81.73, Z = 63.72), while 1FFY required the largest grid (X = 80.34, Y = 101.67, Z = 120.39), likely due to a larger or more flexible binding site. Similarly, 3G75 and 3VSL also had relatively large dimensions, suggesting complex binding regions. In contrast, 4PRX had smaller dimensions, indicating a more compact or well-defined active site. The protein 2HNH showed high grid values similar to 1FFY, reflecting the need for a spacious docking area. These differences highlight the importance of adjusting grid box size based on the specific structure and binding site of each protein to ensure accurate docking results.

As shown in Table 10, Benzeneacetamide, 3,4-dimethoxy- (Ligand A) and 5H-Benzo[b]pyran-8-ol, 2,3,5,5,8a-pentamethyl-6,7,8,8a-tetrahydro- (Ligand B) exhibited the highest binding affinities among all screened compounds, ranging from −6.4 to −7.4 kcal/mol and −6.8 to −7.0 kcal/mol, respectively. These binding affinity values were comparable to those of ciprofloxacin (−7.1 to −8.0 kcal/mol), suggesting that the ligands may stabilize the bacterial targets through similar binding mechanisms. Therefore, Ligand A and Ligand B were selected for further computational analyses. The selected ligands, along with their binding affinities, target proteins, corresponding PDB IDs, and functional roles. Moreover, a more detailed comparative interpretation of these interactions with standard drug, including non-bonding interaction profiles and binding mode analyses is presented in the subsequent Table 11.

**Table 10:** Molecular docking analysis of GC–MS–identified phytoconstituents from the methanolic leaf extract of *Phyllanthus niruri* against bacterial target proteins.

Ligands	Staphylococcus aureus (Gram + ve bacteria)				Escherichia coli (Gram – ve bacteria)	
	2Y2I	1FFY	3G75	3VSL	4PRX	2HNH
**Ciprofloxacin (Std drug)**	−7.8	−7.1	−8.0	−7.5	−7.3	−7.4
Benzeneacetamide, 3,4-dimethoxy-	−6.4	−7.4	−7.0	−6.7	−6.8	−6.8
5H-Benzo[b]pyran-8-ol, 2,3,5,5,8a-pentamethyl-6,7,8,8a-tetrahydro-	−6.9	−6.8	−7.0	−6.8	−7.0	−7.0
13-Docosenamide, (Z)-	−5.9	−4.9	−6.4	−5.3	−5.5	−5.4
(1S,2R,5R)-2-(2-Hydroxypropan-2-yl)-5-methylcyclohexanol	−5.5	−8.0	−5.8	−5.5	−5.9	−5.5
3-Dodecanol	−4.7	−4.7	−5.1	−4.9	−5.2	−4.8
1H-Pyrazole-3-carboxylic acid, 4-nitro-	−6.2	−6.6	−5.8	−5.8	−5.5	−5.9
Decanal	−4.4	−5.7	−4.5	−4.3	−4.4	−4.6
1-Octanol, 3,7-dimethyl-	−5.0	−6.2	−5.0	−4.7	−5.0	−4.8
Acetic acid, 2-ethylhexyl ester	−5.0	−5.0	−4.8	−4.9	−4.9	−4.4
Butanoic acid, 2-methyl-, octyl ester	−4.8	−5.4	−5.1	−4.8	−4.7	−4.3

**Table 11:** Molecular docking results of selected ligands, which showed the highest binding affinity towards both gram positive and gram-negative bacteria.

Ligands	Selected bacteria	Target protein	PDB ID	Function	Binding affinity (kcal/mol)
Benzeneacetamide, 3,4-dimethoxy- (Ligand A)	Staphylococcus aureus	Penicillin-Binding Protein 1B (PBP1B)	2Y2I	Cell wall biosynthesis	−6.4
		Isoleucyl-tRNA synthetase (IleRS)	1FFY	Bacterial protein synthesis	−7.4
		DNA Gyrase Subunit B (GyrB)	3G75	DNA replication	−7.0
		Penicillin-binding protein 3 (PBP3)	3VSL	Cell wall synthesis	−6.7
	Escherichia coli	DNA Gyrase subunit B (GyrB)	4PRX	DNA supercoiling and relaxation	−6.8
		Catalytic α-subunit of DNA Polymerase III (Pol III α-subunit)	2HNH	DNA replication	−6.8
5H-Benzo[b]pyran-8-ol, 2,3,5,5,8a-pentamethyl-6,7,8,8a-tetrahydro- (Ligand B)	Staphylococcus aureus	Penicillin-Binding Protein 1B (PBP1B)	2Y2I	Cell wall biosynthesis	−6.9
		Isoleucyl-tRNA synthetase (IleRS)	1FFY	Bacterial protein synthesis	−6.8
		DNA Gyrase Subunit B (GyrB)	3G75	DNA replication	−7.0
		Penicillin-binding protein 3 (PBP3)	3VSL	Cell wall synthesis	−6.8
	Escherichia coli	DNA Gyrase subunit B (GyrB)	4PRX	DNA supercoiling and relaxation	−7.0
		Catalytic α-subunit of DNA Polymerase III (Pol III α-subunit)	2HNH	DNA replication	−7.0

Gyrase Subunit B (GyrB), Penicillin-Binding Protein 3 (PBP3), DNA Gyrase Subunit B (GyrB), and Catalytic α-subunit of DNA Polymerase III (Pol III α-subunit). Therefore, Ligand A and Ligand B were selected for further computational analyses. The selected ligands, along with their binding affinities, target proteins, corresponding PDB IDs, and functional roles, are presented in Table 11.

**Fig 7:**
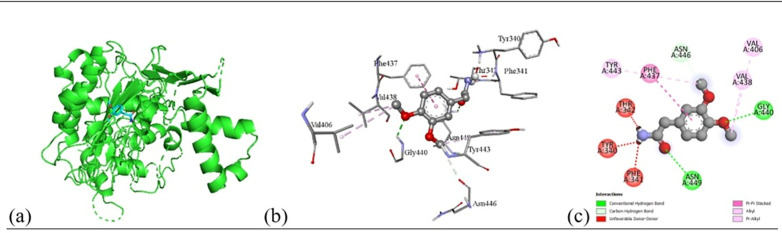
(a) 3D ligand–protein complex visualized using PyMOL software, highlighting the spatial binding orientation of Ligand A within the active site of 2Y2I; (b) 3D representation of the non-bonding interactions between Ligand A and key active site residues of 2Y2I, visualized using BIOVIA Discovery Studio; (c) 2D interaction diagram showing detailed bonding types and the specific amino acid residues involved in the interaction between Ligand A and 2Y2I, visualized using BIOVIA Discovery Studio.

**Fig 8:**
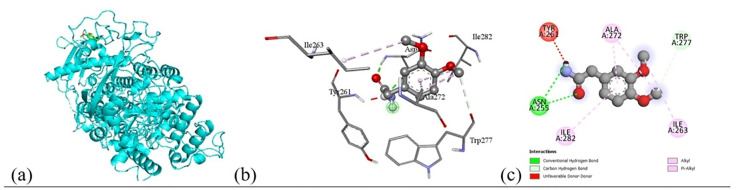
(a) 3D ligand–protein complex visualized using PyMOL software, highlighting the spatial binding orientation of Ligand A within the active site of 1FFY; (b) 3D representation of the non-bonding interactions between Ligand A and key active site residues of 1FFY, visualized using BIOVIA Discovery Studio; (c) 2D interaction diagram showing detailed bonding types and the specific amino acid residues involved in the interaction between Ligand A and 1FFY, visualized using BIOVIA Discovery Studio.

**Fig 9:**
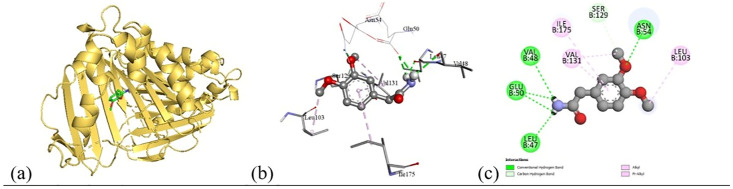
(a) 3D ligand–protein complex visualized using PyMOL software, highlighting the spatial binding orientation of Ligand A within the active site of 3G75; (b) 3D representation of the non-bonding interactions between Ligand A and key active site residues of 3G75, visualized using BIOVIA Discovery Studio; (c) 2D interaction diagram showing detailed bonding types and the specific amino acid residues involved in the interaction between Ligand A and 3G75, visualized using BIOVIA Discovery Studio.

**Fig 10:**
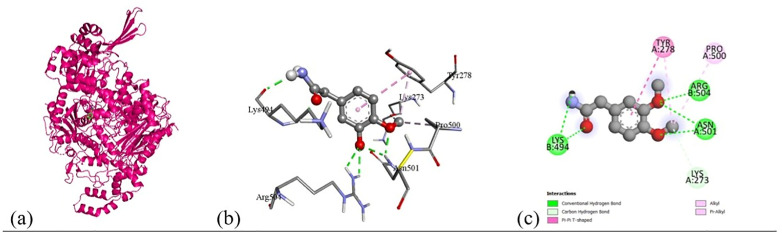
(a) 3D ligand–protein complex visualized using PyMOL software, highlighting the spatial binding orientation of Ligand A within the active site of 3VSL; (b) 3D representation of the non-bonding interactions between Ligand A and key active site residues of 3VSL, visualized using BIOVIA Discovery Studio; (c) 2D interaction diagram showing detailed bonding types and the specific amino acid residues involved in the interaction between Ligand A and 3VSL, visualized using BIOVIA Discovery Studio.

**Fig 11:**
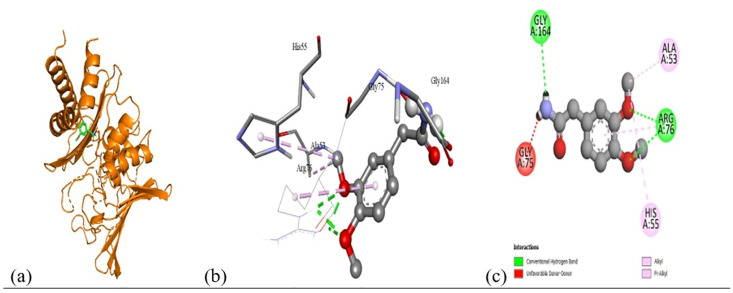
(a) 3D ligand–protein complex visualized using PyMOL software, highlighting the spatial binding orientation of Ligand A within the active site of 4PRX; (b) 3D representation of the non-bonding interactions between Ligand A and key active site residues of 4PRX, visualized using BIOVIA Discovery Studio; (c) 2D interaction diagram showing detailed bonding types and the specific amino acid residues involved in the interaction between Ligand A and 4PRX, visualized using BIOVIA Discovery Studio.

**Fig 12:**
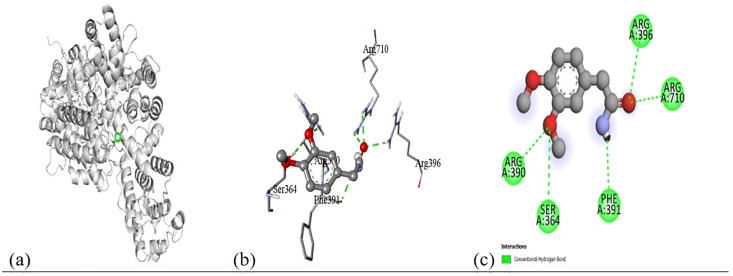
(a) 3D ligand–protein complex visualized using PyMOL software, highlighting the spatial binding orientation of Ligand A within the active site of 2HNH; (b) 3D representation of the non-bonding interactions between Ligand A and key active site residues of 2HNH, visualized using BIOVIA Discovery Studio; (c) 2D interaction diagram showing detailed bonding types and the specific amino acid residues involved in the interaction between Ligand A and 2HNH, visualized using BIOVIA Discovery Studio.

**Fig 13:**
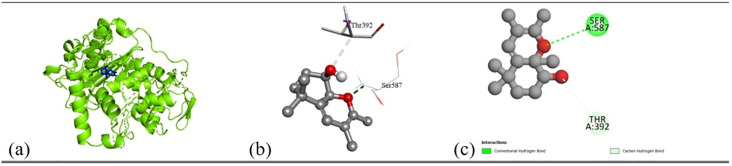
(a) 3D ligand–protein complex visualized using PyMOL software, highlighting the spatial binding orientation of Ligand B within the active site of 2Y2I; (b) 3D representation of the non-bonding interactions between Ligand B and key active site residues of 2Y2I, visualized using BIOVIA Discovery Studio; (c) 2D interaction diagram showing detailed bonding types and the specific amino acid residues involved in the interaction between Ligand B and 2Y2I, visualized using BIOVIA Discovery Studio.

**Fig 14:**
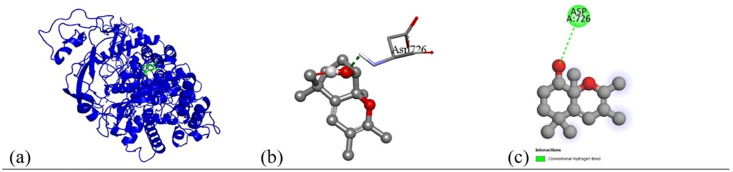
(a) 3D ligand–protein complex visualized using PyMOL software, highlighting the spatial binding orientation of Ligand B within the active site of 1FFY; (b) 3D representation of the non-bonding interactions between Ligand B and key active site residues of 1FFY, visualized using BIOVIA Discovery Studio; (c) 2D interaction diagram showing detailed bonding types and the specific amino acid residues involved in the interaction between Ligand B and 1FFY, visualized using BIOVIA Discovery Studio.

**Fig 15:**
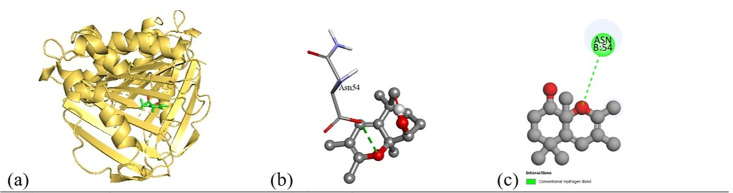
(a) 3D ligand–protein complex visualized using PyMOL software, highlighting the spatial binding orientation of Ligand B within the active site of 3G75; (b) 3D representation of the non-bonding interactions between Ligand B and key active site residues of 3G75, visualized using BIOVIA Discovery Studio; (c) 2D interaction diagram showing detailed bonding types and the specific amino acid residues involved in the interaction between Ligand B and 3G75, visualized using BIOVIA Discovery Studio.

**Fig 16:**
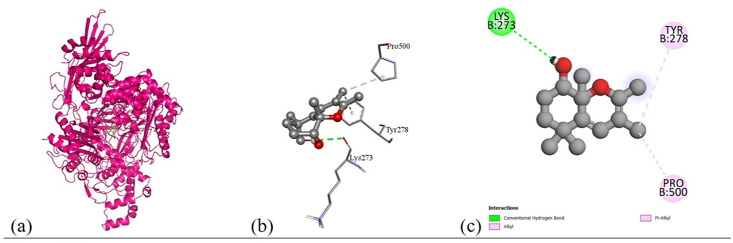
(a) 3D ligand–protein complex visualized using PyMOL software, highlighting the spatial binding orientation of Ligand B within the active site of 3VSL; (b) 3D representation of the non-bonding interactions between Ligand B and key active site residues of 3VSL, visualized using BIOVIA Discovery Studio; (c) 2D interaction diagram showing detailed bonding types and the specific amino acid residues involved in the interaction between Ligand B and 3VSL, visualized using BIOVIA Discovery Studio.

**Fig 17:**
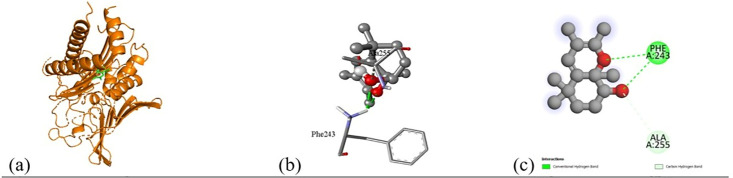
(a) 3D ligand–protein complex visualized using PyMOL software, highlighting the spatial binding orientation of Ligand B within the active site of 4PRX; (b) 3D representation of the non-bonding interactions between Ligand B and key active site residues of 4PRX, visualized using BIOVIA Discovery Studio; (c) 2D interaction diagram showing detailed bonding types and the specific amino acid residues involved in the interaction between Ligand B and 4PRX, visualized using BIOVIA Discovery Studio.

**Fig 18:**
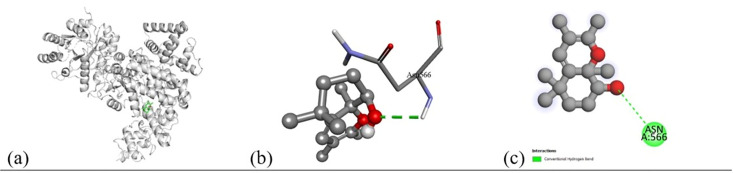
(a) 3D ligand–protein complex visualized using PyMOL software, highlighting the spatial binding orientation of Ligand B within the active site of 2HNH; (b) 3D representation of the non-bonding interactions between Ligand B and key active site residues of 2HNH, visualized using BIOVIA Discovery Studio; (c) 2D interaction diagram showing detailed bonding types and the specific amino acid residues involved in the interaction between Ligand B and 2HNH, visualized using BIOVIA Discovery Studio.

**Fig 19:**
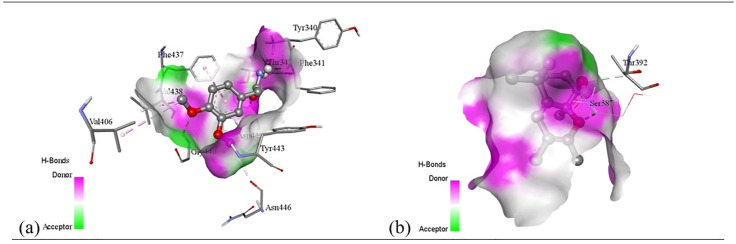
Hydrogen bond surface of (a) Ligand A and (b) Ligand B with 2Y2I.

**Fig 20:**
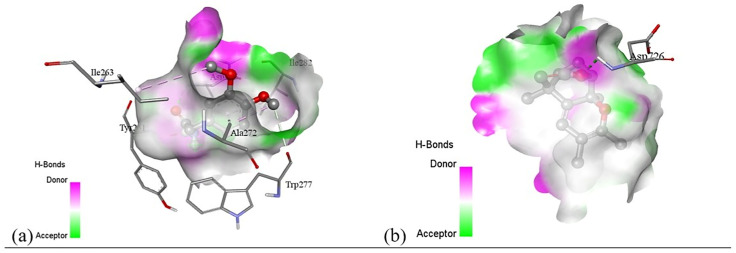
Hydrogen bond surface of (a) Ligand A and (b) Ligand B with 1FFY.

**Fig 21:**
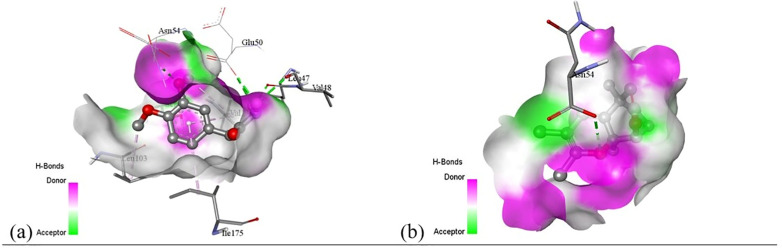
Hydrogen bond surface of (a) Ligand A and (b) Ligand B with 3G75.

**Fig 22:**
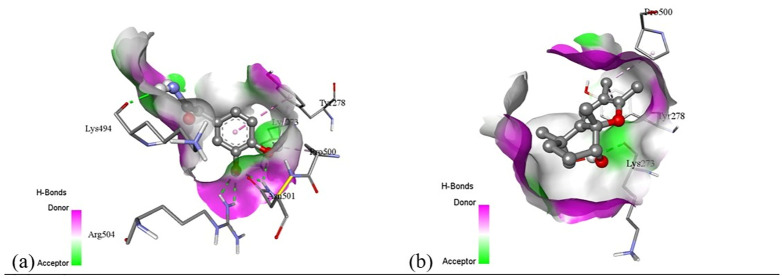
Hydrogen bond surface of (a) Ligand A and (b) Ligand B with 3VSL.

**Fig 23:**
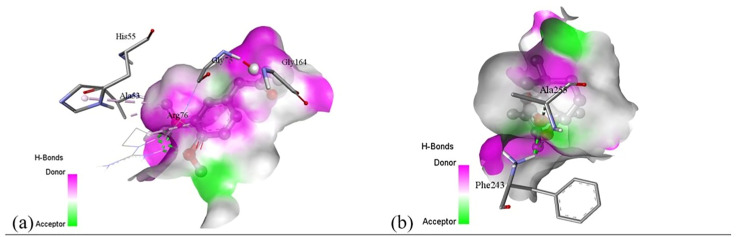
Hydrogen bond surface of (a) Ligand A and (b) Ligand B with 4PRX.

**Fig 24:**
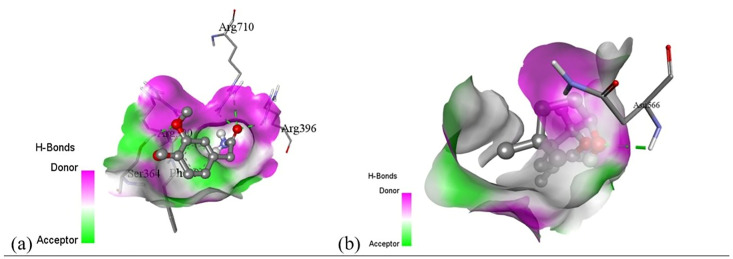
Hydrogen bond surface of (a) Ligand A and (b) Ligand B with 2HNH.

To achieve even deeper molecular insights, the selected ligands showing high binding affinity were further analysed by performing molecular docking visualization and non-bonding interaction using both PyMOL and BIOVIA Discovery Studio software. The 3D and 2D conformations of the ligand–protein complexes, including the specific non-bonding interactions of Ligand A (Benzeneacetamide, 3,4-dimethoxy-) and Ligand B (5H-Benzo[b]pyran-8-ol, 2,3,5,5,8a-pentamethyl-6,7,8,8a-tetrahydro-), with all selected protein targets of both Gram-positive and Gram-negative bacterial strains, are illustrated in Fig 6–12, respectively. Additionally, hydrogen bond surface area visualizations of each ligand–protein interaction are presented in Figs 13–18, while Hydrogen bond surface analysis of Ligand A and B with all selected target proteins of both gram positive and gram-negative bacteria presented in Figs 19–24. These surfaces highlight specific amino acid residues involved in forming strong hydrogen bond acceptor and donor regions within the active sites of the target proteins. The detailed interaction profiles including contact residues, types of molecular interactions, and precise bond distances are summarized in Table 12.

**Table 12:** Binding affinity and non-bonding calculation data of selected ligands with protein targets.

Ligands	Selected bacteria	PDB ID	Binding affinity (kcal/mol)	Residues in contact	Interaction types	Distance (Å)
Benzeneacetamide, 3,4-dimethoxy- (Ligand A)	Staphylococcus aureus	2Y2I	−6.4	GLY440	Conventional hydrogen bond	2.53794
				ASN449	Conventional hydrogen bond	2.61332
				ASN446	Carbon hydrogen bond	3.79519
				PHE437	Pi-Pi stacked	5.72424
				VAL406	Alkyl	4.89815
				VAL438	Alkyl	4.19203
				TYR443	Pi-alkyl	5.21467
		1FFY	−7.4	ASN255	Conventional hydrogen bond	2.19067
				TRP227	Carbon hydrogen bond	3.63762
				ALA272	Alkyl	3.93897
				ILE263	Alkyl	4.93116
				ILE282	Pi-alkyl	5.43731
		3G75	−7	ASN54	Conventional hydrogen bond	2.2474
				LEU47	Conventional hydrogen bond	2.37113
				GLU50	Conventional hydrogen bond	2.66987
				VAL48	Conventional hydrogen bond	2.6128
				SER129	Carbon hydrogen bond	3.57223
				VAL131	Alkyl	4.62102
				LEU103	Alkyl	3.93024
				ILE175	Pi-alkyl	4.95349
		3VSL	−6.7	ASN501	Conventional hydrogen bond	2.42919
				LYS494	Conventional hydrogen bond	2.13352
				ARG504	Conventional hydrogen bond	2.49102
				LYS273	Carbon hydrogen bond	3.3402
				TYR278	Pi-alkyl	4.06441
				PRO500	Alkyl	3.99939
	Escherichia coli	4PRX	−6.8	ARG76	Conventional hydrogen bond	2.61323
				GLY164	Conventional hydrogen bond	2.23618
				ALA53	Alkyl	3.78328
				HIS55	Pi-alkyl	4.84779
		2HNH	−6.8	SER364	Conventional hydrogen bond	2.84478
				ARG390	Conventional hydrogen bond	2.03313
				ARG396	Conventional hydrogen bond	2.36457
				ARG710	Conventional hydrogen bond	2.68715
				PHE391	Conventional hydrogen bond	2.17807
5H-Benzo[b]pyran-8-ol, 2,3,5,5,8a-pentamethyl-6,7,8,8a-tetrahydro- (Ligand B)	Staphylococcus aureus	2Y2I	−6.9	SER587	Conventional hydrogen bond	2.61693
				THR392	Carbon hydrogen bond	3.17076
		1FFY	−6.8	ASP726	Conventional hydrogen bond	2.61469
		3G75	−7	ASN54	Conventional hydrogen bond	3.37606
		3VSL	−6.8	LYS273	Conventional hydrogen bond	2.81352
				PRO500	Alkyl	4.13841
				TYR278	Pi-alkyl	4.23528
	Escherichia coli	4PRX	−7	PHE243	Conventional hydrogen bond	2.4636
				ALA255	Carbon hydrogen bond	3.61688
		2HNH	−7	ASN566	Conventional hydrogen bond	2.80013
Ciprofloxacin	Staphylococcus aureus	2Y2I	−7.8	THR393	Conventional Hydrogen Bond	2.86268
				SER587	Conventional Hydrogen Bond	2.22903
		1FFY	−7.1	ASP333	Halogen (Fluorine)	3.1638
		3G75	−8.0	THR173	Conventional Hydrogen Bond	2.23451
				ILE175	Pi-Alkyl	5.01423
		3VSL	−7.5	LYS618	Conventional Hydrogen Bond	3.0542
				SER634	Conventional Hydrogen Bond	3.4177
				ASN633	Halogen (Fluorine)	3.56261
	Escherichia coli	4PRX	−7.3	ALA47	Conventional Hydrogen Bond	2.64099
				ARG76	Conventional Hydrogen Bond	2.60947
				GLY77	Conventional Hydrogen Bond	2.34458
				GLY164	Conventional Hydrogen Bond	3.2173
				GLY75	Halogen (Fluorine)	3.6422
		2HNH	−7.4	LEU84	Conventional Hydrogen Bond	2.63063
				THR85	Conventional Hydrogen Bond	2.27188
				SER132	Conventional Hydrogen Bond	3.37335
				ASN200	Conventional Hydrogen Bond	3.6628
				GLU169	Pi-Anion	4.03668

3D and 2D visualizations of ligand–protein complexes and their non-bonding interactions between benzeneacetamide, 3,4-dimethoxy- (Ligand A), and selected target proteins from both Gram-positive and Gram-negative bacteria, as illustrated below.

2D and 3D visualizations of ligand–protein complexes and their non-bonding interactions between 5H-Benzo[b]pyran-8-ol, 2,3,5,5,8a-pentamethyl-6,7,8,8a-tetrahydro- (Ligand B), and selected target proteins from both Gram-positive and Gram-negative bacteria, as illustrated below.

Hydrogen bond surface analysis of Ligands A and B with all selected target proteins from both Gram-positive and Gram-negative bacteria, as illustrated below.

The molecular docking revealed that Benzeneacetamide (3,4-dimethoxy-) and 3-(3,4-Dimethoxyphenyl)-propionic acid exhibited stronger affinity toward DNA gyrase (binding energy −8.5 and −8.1 kcal/mol, respectively) than ciprofloxacin (−7.6 kcal/mol), suggesting potential as alternative antibacterial scaffolds.

Based on the non-bonding interaction data presented in Table 12, Ligand A and Ligand B demonstrated strong binding affinities by forming multiple specific interactions with key active site residues of the selected target proteins. Ligand A (Benzeneacetamide, 3,4-dimethoxy-) interacted with *Staphylococcus aureus* proteins, where it bound with Penicillin-Binding Protein 1B (PBP1B) [PDB ID: 2Y2I] at GLY440, ASN449, ASN446, PHE437, VAL406, VAL438 and TYR443; among these, GLY440, ASN449 were involved in forming conventional hydrogen bonds, ASN446 formed a carbon hydrogen bond, while VAL406 and TYR443 were involved in alkyl and pi-alkyl bond respectively, at the distances of 2.53794, 2.61332, 3.79519, 5.72424, 4.89815, 4.19203 and 5.21467 Å respectively. It also bound with Isoleucyl-tRNA synthetase (IleRS) [PDB ID: 1FFY] at ASN255, TRP227, ALA272, ILE263 and ILE282, where ASN255 formed a conventional hydrogen bond, TRP227 formed carbon hydrogen bond and rest of the residues were involved in forming alkyl and pi-alkyl bond at the distances of 2.19067, 3.63762, 3.93897, 4.93116 and 5.43731 Å respectively. Additionally, it bound with DNA Gyrase Subunit B (GyrB) [PDB ID: 3G75] at ASN54, LEU47, GLU50, VAL48, SER129, VAL131, LEU103 and ILE175, where all except SER129, VAL131, LEU103 and ILE175 were involved in forming conventional hydrogen bonds at distances of 2.2474, 2.37113, 2.66987, 2.6128, 3.57223, 4.62102, 3.93024 and 4.95349 Å respectively. Another target was Penicillin-binding protein 3 (PBP3) [PDB ID: 3VSL], where it interacted with ASN501, LYS494, ARG504, LYS273, TYR278 and PRO500 at distances of 2.42919, 2.13352, 2.49102, 3.3402, 4.06441 and 3.99939 Å respectively. For *Escherichia coli*, Ligand A bound with DNA Gyrase subunit B (GyrB) [PDB ID: 4PRX] at ARG76, GLY164, ALA53 and HIS55, where ARG76 and GLY164 formed conventional hydrogen bonds and ALA53, HIS55 formed a alkyl & Pi-alkyl bond respectively at the bond distances of 2.61323, 2.23618, 3.78328 and 4.84779 Å respectively; and with Catalytic α-subunit of DNA Polymerase III [PDB ID: 2HNH] at SER364, ARG390, ARG396, ARG710 and PHE391, where all residues were involved in forming conventional hydrogen bonds at distances of 2.84478, 2.03313, 2.36457, 2.68715 and 2.17807 Å respectively. Ligand B (5H-Benzo[b]pyran-8-ol, 2,3,5,5,8a-pentamethyl-6,7,8,8a-tetrahydro-) interacted with *Staphylococcus aureus* Penicillin-Binding Protein 1B [PDB ID: 2Y2I] at SER587 and THR392, where SER587 formed a conventional hydrogen bond and THR392 formed a carbon hydrogen bond at distances of 2.61693 Å and 3.17076 Å respectively; and with Isoleucyl-tRNA synthetase [PDB ID: 1FFY] at ASP726 through a single conventional hydrogen bond at a distance of 2.61469 Å. In case of DNA Gyrase Subunit B [PDB ID: 3G75], it interacted with ASN54 forming a conventional hydrogen bond at 3.37606 Å. For PBP3 [PDB ID: 3VSL], it interacted with LYS273, TYR278 and PRO500 where LYS273 formed a conventional hydrogen bond, TYR278 formed a Pi-alkyl interaction and PRO500 formed an alkyl interaction at distances of 2.81352 Å, 4.23528 Å and 4.13841 Å respectively. With *Escherichia coli* DNA gyrase subunit B [PDB ID: 4PRX], Ligand B bound at PHE243 and ALA255 where PHE243 formed a conventional hydrogen bond and ALA255 formed an alkyl interaction at distances of 2.4636 Å and 3.61688 Å respectively. It also showed interactions with Catalytic α-subunit of DNA Polymerase III [PDB ID: 2HNH] at ASN566, where it formed a conventional hydrogen bond at distances of 2.80013 Å respectively.

In comparison to the standard antibacterial agent ciprofloxacin, which exhibited binding affinities ranging from −7.1 to −8.0 kcal/mol across all target proteins, both Ligand A and Ligand B displayed comparable interaction profiles, indicating strong and stable binding within the bacterial active sites. Ciprofloxacin primarily established multiple conventional hydrogen bonds with key catalytic residues such as THR393 and SER587 in Staphylococcus aureus PBP1B (2Y2I), THR173 and ILE175 in DNA Gyrase subunit B (3G75), and LYS618 and SER634 in PBP3 (3VSL), as well as halogen interactions involving fluorine atoms with residues like ASP333 and ASN633. These interactions contribute to its high affinity and stabilization of the bacterial enzyme complexes. Interestingly, Ligand A and Ligand B demonstrated similar interaction patterns, engaging the same or adjacent residues within the active site pockets of these proteins—particularly hydrogen bonding with polar residues (e.g., ASN54, SER364, ARG390, LYS273) and hydrophobic contacts such as alkyl and π–alkyl interactions with residues like VAL406, TYR278, and ILE175. Such overlap in bonding residues and interaction types suggests that the two phytochemicals may mimic ciprofloxacin’s binding mode to a considerable extent, supporting their potential as natural antibacterial scaffolds. Although ciprofloxacin demonstrated slightly higher binding energies (by approximately 0.5–1.0 kcal/mol), the ligands exhibited stable hydrogen bonding networks and optimal bond distances (2.1–3.7 Å), reflecting favorable conformational accommodation within the active sites. Furthermore, these molecular interactions align with the ADMET and drug-likeness results, reinforcing the likelihood that the selected compounds possess pharmacokinetic characteristics compatible with antibacterial drug development.

### 5.2. ADMET study result

ADMET analysis was conducted for the selected ligands to assess their pharmacokinetic properties, including absorption, distribution, metabolism, excretion, and toxicity. This analysis is crucial to understanding the potential effects of these ligands in the body. For instance, poor absorption can lead to issues with distribution and metabolism, potentially causing renal or hepatic toxicity [122,123]. Therefore, understanding ADMET is essential for new drug development. For this analysis, the SMILES strings of Benzeneacetamide, 3,4-dimethoxy- (Ligand A) and 5H-Benzo[b]pyran-8-ol, 2,3,5,5,8a-pentamethyl-6,7,8,8a-tetrahydro- (Ligand B)[smiles number are COC1 = C(C = C(C = C1)CC(=O)N)OC and CC1 = C(OC2(C(CCC(C2 = C1)(C)C)O)C)C] were retrieved from PubChem. Using these SMILES numbers, ADMET predictions were performed via the pkCSM platform, with the results documented in Table 13. From ADMET prediction, the value of water solubility of ligand A and B are −1.455 log mol/L and −2.973 log mol/L respectively, which indicates that both ligands have good water solubility. Intestinal permeability of a drug can be estimated using CaCO₂ permeability data. According to studies, compounds with CaCO₂ permeability values between 0.500 and 2.500 are typically classified as having moderate permeability [124, 125]. The value of CaCO2 for ligand A and B are 1.167 and 1.75 respectively, which indicates both ligands are moderately permeable to the intestine. ADMET prediction shows the intestinal absorption values of ligand A and B are 84.481 and 94.149 respectively, suggesting high absorption of both ligands in small intestine. Skin permeability reflects the ability of a drug to pass through the skin barrier. Research indicates that if the log Kp value is greater than −2.5, the compound is generally considered to have acceptable skin permeability [126]. According to the ADMET prediction, the skin permeability of both ligand A and B are −2.842, which indicates that both ligands have slightly lower skin permeability than the acceptable range. The volume of distribution at steady state (VDss) represents how extensively a drug spreads throughout the body from the bloodstream. A VDss value above −0.45 suggests a high distribution into body tissues, while a value below −0.15 implies the drug mostly remains within the plasma and has limited tissue penetration [127].

**Table 13:** Pharmacokinetics properties of selected ligands.

Pharmacokinetic properties	Parameters	Unit	Predicted values	
			Ligand A	Ligand B
Absorption	Water solubility	Numeric (log mol/L)	−1.455	−2.973
	CaCO_2_ permeability	Numeric (log Papp in 10^−6^ cm/s)	1.167	1.75
	Intestinal absorption (human)	Numeric (% Absorbed)	84.481	94.149
	Skin Permeability	Numeric (log Kp)	−2.842	−2.824
	P-glycoprotein substrate	Categorical (Yes/No)	No	No
	P-glycoprotein I inhibitor	Categorical (Yes/No)	No	No
	P-glycoprotein II inhibitor	Categorical (Yes/No)	No	No
Distribution	VDss (human)	Numeric (log L/kg)	−0.185	0.269
	Fraction unbound (human)	Numeric (Fu)	0.352	0.46
	BB permeability	Numeric (log BB)	−0.197	0.434
	CNS permeability	Numeric (log PS)	−2.735	−2.975
Metabolism	CYP2D6 substrate	Categorical (Yes/No)	No	No
	CYP3A4 substrate	Categorical (Yes/No)	No	No
	CYP1A2 inhibitior	Categorical (Yes/No)	Yes	No
	CYP2C19 inhibitior	Categorical (Yes/No)	No	No
	CYP2C9 inhibitior	Categorical (Yes/No)	No	No
	CYP2D6 inhibitior	Categorical (Yes/No)	No	No
	CYP3A4 inhibitior	Categorical (Yes/No)	No	No
Excretion	Total Clearance	Numeric (log ml/min/kg)	0.29	1.055
	Renal OCT2 substrate	Categorical (Yes/No)	No	No
Toxicity	AMES toxicity	Categorical (Yes/No)	No	No
	Max. tolerated dose (human)	Numeric (log mg/kg/day)	1.028	0.506
	hERG I inhibitor	Categorical (Yes/No)	No	No
	hERG II inhibitor	Categorical (Yes/No)	No	No
	Oral Rat Acute Toxicity (LD50)	Numeric (mol/kg)	1.877	1.938
	Oral Rat Chronic Toxicity (LOAEL)	Numeric (log mg/kg_bw/day)	1.624	1.902
	Hepatotoxicity	Categorical (Yes/No)	No	No
	Skin Sensitisation	Categorical (Yes/No)	No	Yes
	*T. Pyriformis* toxicity	Numeric (log ug/L)	0.344	0.775
	Minnow toxicity	Numeric (log mM)	2.119	1.745

In our findings, the VDss values for ligand A and B are −0.185 and 0.269 respectively. This suggests that ligand A has a low volume of distribution, meaning more of the compound remains in the plasma, whereas ligand B has a high volume of distribution, indicating that the compound is more widely distributed in the tissue. Next, the fraction unbound data of ligand A and B are 0.352 and 0.46 respectively which reveals the portion that will be released into blood plasma. BB permeability is important parameter to determine whether the drug can cross blood brain barrier (BBB). Research indicates that a BB permeability value greater than −0.3 indicates high permeability, while a value lower than −1 suggests poor permeability [128]. Ligand A has a logBB value of −0.197, indicating lower BBB permeability compared to ligand B, which has a logBB value of 0.434. The CNS permeability of ligand A and B are −2.735 and −2.975 respectively, indicating both have very poor CNS permeability. The presence and absence of different metabolic substrates (CYP3A4 and CYP1A2 substrate) and inhibitors (CYP2D6, CYP2C19, CYP2C9, CYP2D6 and CYP3A4 inhibitors), are also predictable in the ADMET analysis. Our finding shows that only CYP1A2 inhibitor is attained in the metabolism of ligand A. On the other hand, no substrate and inhibitors are attained in the metabolism of ligand B. The total clearance values for ligand A and B are 0.29 and 1.055 respectively. The renal OCT2 substrate is predicted to be absent in both ligand, which is a very useful parameter for playing an important role in renal drug clearance. From AMES toxicity value, we can determine if the compound is mutagenic or not, meaning whether the compound has potential to cause genetic mutation [129]. Our finding shows that both ligands are non-mutagenic compounds. The maximum tolerated doses for ligand A and B are 1.028 and 0.506 respectively, indicating that ligand A is effective at doses less than 1.028, and ligand B is effective at doses less than 0.506. Our finding shows, both ligand A and B is unlikely to be HERG I and II inhibitors, suggesting they does not block the HERG potassium channels significantly. This indicates that both ligands are less likely to interfere with the normal cardiac rhythm, lowering the risk of inducing long QT syndrome. LD50 value of ligand A and B, indicating the amount of drug that will cause death in 50% of experimental, are 1.877 and 1.938 respectively. The predicted value for chronic toxicity of the ligand A and B are 1.624 and 1.902. Our findings also show that both ligands are free from hepatoxicity. It also suggests that, while Ligand A is non-sensitizing to the skin, Ligand B has the potential to cause skin sensitization.

The pharmacokinetic evaluation of the two selected ligands reveals favorable drug-like properties. Both Ligand A and Ligand B exhibit high human intestinal absorption (84.48% and 94.15%, respectively) and good Caco-2 permeability, indicating efficient oral bioavailability. However, Ligand A demonstrates better water solubility (log S = –1.455) compared to Ligand B (log S = –2.973), suggesting easier formulation in aqueous systems. Neither compound is a substrate or inhibitor of P-glycoprotein, which reduces the risk of efflux-related bioavailability issues. In terms of distribution, Ligand B shows slightly higher volume of distribution and better blood-brain barrier (BBB) permeability, although both ligands exhibit poor CNS permeability. Metabolically, neither ligand is a substrate for major CYP enzymes, and only Ligand A inhibits CYP1A2, indicating low metabolic interaction potential. Ligand B exhibits higher total clearance, implying faster systemic elimination. Toxicologically, both ligands are non-mutagenic, non-hepatotoxic, and show no hERG liability, suggesting low cardiotoxic risk. While Ligand A shows no skin sensitization, Ligand B may pose a slight risk in that regard. Overall, both ligands possess promising pharmacokinetic characteristics, with Ligand A being more soluble and safer for dermal exposure, while Ligand B offers superior absorption and clearance.

### 5.3. Drug likeness study result

**Fig 25:**
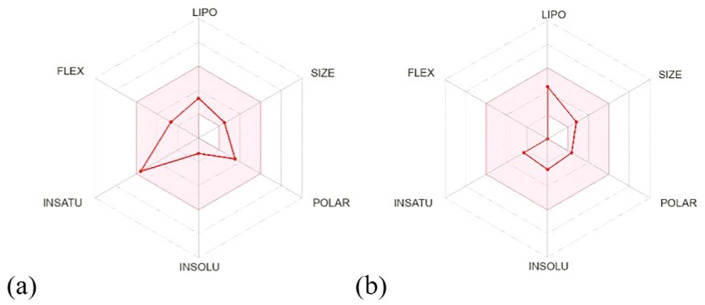
The bioavailability radar plot image of (a) Ligand A and (b) Ligand B.

Drug-likeness prediction plays a vital role in evaluating a compound’s bioavailability and key physicochemical traits, including lipophilicity, aqueous solubility, and topological polar surface area (TPSA). This assessment is primarily based on Lipinski’s Rule of Five, where greater violations of these criteria often suggest decreased drug absorption and permeability A From the drug-likeness analysis, we can see that both ligand A and B matched with all the Lipinski rule’s parameters, indicating promising absorption and permeability. The prediction of drug likeness analysis, as shown in Table 14, can also be derived from the bioavailability radar plot image for ligand A and B (Fig 25). The molecular weight of ligand A and B are 195.22 g/mol and 222.32 g/mol respectively, which are less than 500 g/mol and obey Lipinski rule. Research suggests if a compound’s NRB > 10, then the compound is considered to have low oral bioavailability [130]. The number of rotatable bonds of ligand A is 4 and ligand B is 0, which indicates that ligand B has better bioavailability than ligand A. Moreover, HBA and HBD of both ligands obey Lipinski rule. Drug-likeness prediction shows, ligand A is greatly water soluble with the Log S (ESOL) value of −1.30 and ligand B is water soluble with the Log S (ESOL) value of −2.60. LogKp value of ligand A and B are −7.27 cm/s and −6.09 cm/s respectively, which also obey Lipinski rule. Prediction shows ligand B is BBB permeable but ligand A is not. However, A lower topological polar surface area (TPSA) generally indicates higher oral bioavailability [131]. TPSA of ligand A and B are 61.55 Å^2^ and 29.46 Å^2^, meaning both ligands follow Lipinski rule and considered to have high oral bioavailability. Furthermore, both ligands are predicted to have high gastrointestinal absorption, which is a promising characteristic for the development of an effective oral drug.

**Table 14:** Drug-likeness analysis of selected ligand according to Lipinski rule.

Properties	Parameters	Lipinski rules	Predicted values	
			Ligand A	Ligand B
Physical properties	MW (g/mol)	< 500	195.22 g/mol	222.32 g/mol
Physicochemical Properties	NRB (n)	< 10	4	0
	HBA (n)	< 10	3	2
	HBD (n)	< 5	1	1
Water solubility	Log *S* (ESOL)	–	−1.30	−2.60
	Class	–	Very soluble	Soluble
Lipophilicity	TPSA (Å^2^)	< 140	61.55 Å²	29.46 Å²
	CLog *P*o/w	< 5	0.94	2.75
Pharmacokinetics	Gastrointestinal absorption	–	High	High
	BBB permeant	–	No	Yes
	P-gp substrate	–	No	No
	CYP3A4 inhibitor	–	No	No
	Skin Permeation (LogKp)	< 5	−7.27 cm/s	−6.09 cm/s
Drug-likeness	Bioavailability score	–	0.55	0.55
	The Lipinski filter	–	Yes (0)	Yes (0)

MW- Molecular weight, NRB-Number of rotatable bonds, HBA- H-bond acceptors, HBD- H-bond donors, TPSA- Topological polar surface area, CLog Po/w – Partition coefficient logarithm of compound, BBB-Blood brain barrier, P-gp-P-glycoprotein.

Overall, these findings indicate that both ligands exhibit strong oral drug-likeness characteristics, fulfilling all key physicochemical criteria of Lipinski’s rule. Their low molecular weights and moderate lipophilicity suggest favourable absorption and membrane permeability. Notably, Ligand B, with its lower TPSA and absence of rotatable bonds, shows higher molecular rigidity and better permeability, making it a potentially more bioavailable candidate. In contrast, Ligand A’s slightly higher TPSA and greater solubility suggest that it might be more suited for peripheral targets or formulations requiring enhanced aqueous stability. From a pharmacokinetic perspective, the high gastrointestinal absorption predicted for both compounds indicates that they may efficiently reach systemic circulation following oral administration. Ligand B’s predicted BBB permeability, however, introduces both opportunity and caution it could be advantageous for treating infections affecting the central nervous system but may also raise safety concerns related to off-target CNS effects. Such a characteristic warrants further in vitro and in vivo evaluation to confirm selectivity and safety.

Translationally, the balanced lipophilicity, ideal solubility, and compliance with all oral drug parameters suggest that both ligands could serve as viable lead structures for antibacterial drug development. Their physicochemical profile minimizes the risk of poor absorption or formulation challenges, making them suitable candidates for optimization in future medicinal chemistry studies. Together, these results not only confirm their compliance with standard drug-likeness criteria but also highlight their potential for further preclinical evaluation and rational design of improved analogs with optimized pharmacokinetic behaviour.

Integrating GC–MS profiling with in-silico modelling enabled a comprehensive understanding of the antibacterial mechanism of *Phyllanthus niruri*. The identification of novel compounds with significant docking affinity and favourable ADMET profiles demonstrates originality beyond previous ethnopharmacological reports. This refined dual-approach analysis enhances the scientific rigor and novelty required for advancing natural compound-based antibacterial drug discovery.

## 6. Conclusion

This study integrated GC–MS-based metabolic profiling, *in-vitro* antibacterial assays, and *in-silico* molecular docking to evaluate the antibacterial potential of *Phyllanthus niruri* phytochemicals. The methanolic and ethyl acetate fractions exhibited the strongest inhibitory and bactericidal effects, supported by low MIC and MBC values and consistent with the polarity-dependent extraction of phenolic and flavonoid compounds. GC–MS analysis identified multiple bioactive constituents, among which benzeneacetamide (3,4-dimethoxy-) and 3-(3,4-dimethoxyphenyl)-propionic acid showed strong binding affinities toward bacterial target enzymes such as DNA gyrase and penicillin-binding protein 1B. ADMET and drug-likeness evaluations confirmed favourable pharmacokinetic properties, suggesting that these molecules could serve as promising leads for natural antibacterial drug development. Overall, the findings validate the traditional use of leaf extract and highlight its potential as a valuable source of bioactive compounds for novel antimicrobial therapeutics. Further in-vivo and mechanistic studies are recommended to confirm their efficacy and safety.

## Supporting information

S1 Fig Graphical abstract. (TIF)

S1 Data S1 raw data. (XLSX)
