# Supplementary figures and images for: GC-MS based bioactive profiling of Phyllanthus niruri and its antibacterial potential through experimental and computational studies
Source: PLoS One. 2026 Jan 23;21(1):e0340866. doi: 10.1371/journal.pone.0340866 (PMC12829960; doi:10.1371/journal.pone.0340866)

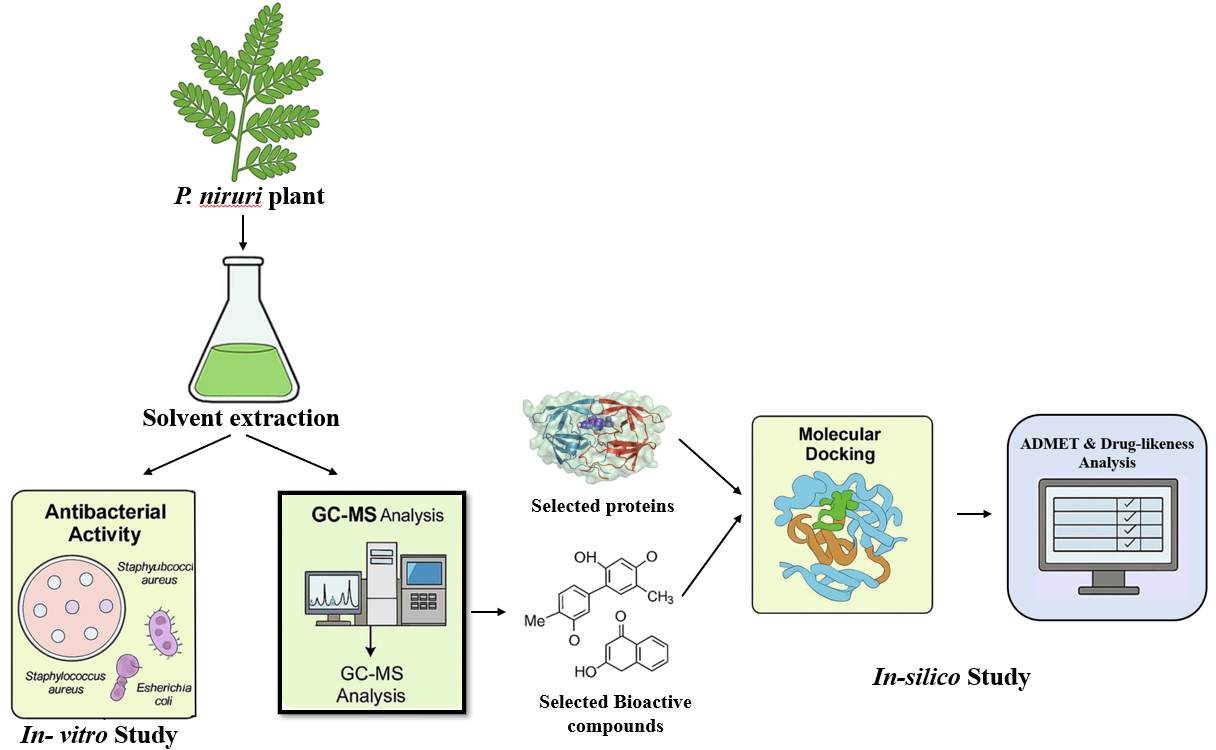

Supplement: S1 Fig — (TIF) [file pone.0340866.s001.tif]
